# Species diversity, taxonomy, multi-gene phylogeny, and divergence times of Meruliaceae (Polyporales, Basidiomycota)

**DOI:** 10.1080/21501203.2024.2443216

**Published:** 2025-01-11

**Authors:** Yue Li, Yi-Feng Cao, Karen K. Nakasone, Shi-Liang Liu, Man-Rong Huang, Shuang-Hui He

**Affiliations:** a Institute of Microbiology, School of Ecology and Nature Conservation, Beijing Forestry University, Beijing, China; b Center for Forest Mycology Research, Northern Research Station, U.S. Forest Service, Madison, WI, USA; c State Key Laboratory of Mycology, Institute of Microbiology, Chinese Academy of Sciences, Beijing, China; d Department of Life Sciences, Natural History Museum of China, Beijing, China

**Keywords:** 24 new taxa, corticioid fungi, molecular clock, phlebioid clade, white rot, wood-decaying fungi

## Abstract

Meruliaceae is one of the three major families of the phlebioid clade in the Polyporales that consists primarily of wood-decaying species. We undertook an in-depth survey on species diversity, generic delimitations, phylogeny, and divergence times within the Meruliaceae with an emphasis on specimens from East Asia. In total, 26 genera including two new genera, *Meruliella* and *Porophlebia*, are recognised; ten new species, viz. *Crustodontia vietnamensis*, *Luteochaete odontoidea*, *Meruliella hainanensis*, *Merulius pinicola*, *Mycoacia beijingensis*, *Phlebiporia crystallifera*, *P. odontoidea*, *Pseudophlebia vesiculosa*, *Scopuloides ellipsoidea*, and *S. grandinioides* are introduced; eleven new combinations, viz. *Allophlebia formosana*, *Aurantiopileus albidus*, *A*. *semisupina*, *Meruliella lindtneri*, *Merulius croceum*, *M. leptospermi*, *M. serialis*, *Phlebicolorata austroasiana*, *Phlebiodontia caspica*, *P. fissurata* and *Porophlebia fimbriata*, and one new name, *Mycoacia neotuberculata*, are proposed. *Noblesia* is placed as a synonymy of *Merulius*, whereas *Ceriporiopsis* and *Lilaceophlebia* are accepted as synonyms of *Mycoacia*. Descriptions and illustrations are provided for the new genera and species, and discussions are provided for all 26 genera and new taxa. The molecular clock analysis results show that the Meruliaceae emerged with a mean stem age of 186.71 Mya of the early Jurassic, and the genera diverged with a mean stem age between 44.29 to 169.46 Mya.

## 1. Introduction

Species in the phlebioid clade of the Polyporales are wood-decaying fungi associated with a white rot of wood and most develop crust-like or corticioid basidiomes with smooth or variously shaped hymenophores (Chen et al. 2021). Within this clade, three well-supported subclades are recognised, represented by the families Phanerochaetaceae, Irpicaceae, and Meruliaceae. The ecology, species diversity, taxonomy and molecular systematics of taxa in these families have been extensively studied (Floudas and Hibbett 2015; Miettinen et al. 2016; Justo et al. 2017; Zmitrovich 2018; Chen et al. 2021; Li et al. 2022, 2023; Lira et al. 2022; Motato-Vásquez et al. 2022; Wang et al. 2023; Yuan et al. 2023; Zhao et al. 2023; Xu et al. 2024). Meruliaceae was introduced by Rea in 1922 and was strongly supported as a monophyletic clade in Polyporales in previous studies (Floudas and Hibbett 2015; Justo et al. 2017; Chen et al. 2021). This family contains a large group of wood-inhabiting fungi with varied macro- and micro-morphological characters. Some of the characters used to identify Meruliaceae taxa including the often ceraceous to gelatinous basidiomes that become crustaceous to corneous after drying, a monomitic hyphal system with rare exceptions, thin-walled, smooth, colourless, small ellipsoid to subcylindrical basidiospores that do not react in Melzer’s reagent or cotton blue, and association with a white rot of angiospermous and coniferous wood. Phylogenetically, many genera are well established and readily recognised within the family, however, the delimitations of some lineages are tenuous because of the low support values of branches and insufficient sampling.

To date, no mushrooms forming, clavarioid, gasteroid or jelly fungi have been found in the family. Meruliaceae contains mostly corticioid and poroid species with a wide range of ecological distribution. The species usually grow on dead standing trees, stumps, fallen trunks, or dead branches of angiosperms, with relatively small portions on gymnosperms, causing a white rot on wood. They play important roles in the energy and material cycles, and a few have economic values, such as *Merulius tremellosus* Fr. can be used for antitumor and antibacterial therapy (Dai et al. 2009), and *Phlebia acanthocystis* Gilb. & Nakasone and *Phlebia brevispora* Nakasone have potential in industrial applications for biodegradation and bioconversion of endrin (Xiao and Kondo 2019). European and North American countries have a long study history of this family, however, in the last few years many new taxa have been described from China (Liu et al. 2020; Chen et al. 2021; Zhao et al. 2023).

So far, 28 genera have been described in Meruliaceae viz. *Allophlebia* C.R.S. de Lira, Gibertoni & K.H. Larss., *Aurantiopileus* Ginns, D.L. Lindner & T.J. Baroni, *Aurantiporus* Murrill, *Ceriporiopsis* Domański, *Ceriporiopsoides* C.L. Zhao, *Climacodon* P. Karst., *Crustodontia* Hjortstam & Ryvarden, *Geesterania* Westphalen, Tomšovský & Rajchenberg, *Hermanssonia* Zmitr., *Hydnophanerochaete* Sheng H. Wu & Che C. Chen, *Hydnophlebia* Parmasto, *Lilaceophlebia* (Parmasto) Spirin & Zmitr., *Luteochaete* C.C. Chen & Sheng H. Wu, *Luteoporia* F. Wu, Jia J. Chen & S.H. He, *Merulius* Fr., *Mycoacia* Donk, *Mycoaciella* J. Erikss. & Ryvarden, *Noblesia* Nakasone, *Odoria* V. Papp & Dima, *Pappia* Zmitr., *Phlebia* Fr., *Phlebicolorata* C.L. Zhao, *Phlebiodontia* Motato-Vásq. & Westphalen, *Phlebiporia* Jia J. Chen, B.K. Cui & Y.C. Dai, *Pseudophlebia* C.L. Zhao, *Sarcodontia* Schulzer, *Scopuloides* (Massee) Höhn. & Litsch., and *Stereophlebia* Zmitr. Genera such as *Allophlebia*, *Luteochaete*, *Noblesia*, *Phlebiodontia*, and *Pseudophlebia* were described recently based on molecular evidence (Chen et al. 2021; Nakasone et al. 2021; Lira et al. 2022; Motato-Vásquez et al. 2022; Zhao et al. 2023). In addition, a few genera such as *Allophlebia*, *Hydnophlebia*, and *Ceriporiopsoides* which were segregated from *Phlebia* sensu lato, *Phanerochaete* s.l., and *Ceriporiopsis* s.l. share similar morphologies, but mostly *Allophlebia* has smooth hymenophores, *Hydnophlebia* has hydnoid hymenophores, and *Ceriporiopsoides* has poroid hymenophores (Telleria et al. 2017; Lira et al. 2022; Zhao et al. 2023). Some genera, such as *Crustodontia*, *Hydnophlebia*, and *Scopuloides*, formed strongly supported, monophyletic clades, whereas several genera, such as *Mycoacia* and *Phlebia* s.s., are not fully resolved. Furthermore, these genera were described based on limited sampling and phylogenetic analyses (Nakasone et al. 2021; Zhao et al. 2023).

Although the family has been relatively intensively studied, several aspects need to be further investigated. The species diversity in some biodiversity hotspots is not fully understood. The phylogenetic placement of some taxa, especially old large genera such as *Merulius*, *Mycoacia*, and *Phlebia* or newly built small genera such as *Noblesia*, *Phlebicolorata* and *Pseudophlebia*, are not clear and taxonomic status of some species including *Ceriporiopsis fimbriata* C.L. Zhao & Y.C. Dai, *Phlebia formosana* Sheng H. Wu and *Sistotrema croceum* Schwein. needs to be re-evaluated.

In this study, we conducted a survey on species diversity, generic delimitations, phylogeny, and divergence times of the Meruliaceae based on a comprehensive sampling including additional specimens from East Asia, viz. China, Malaysia, Sri Lanka, and Vietnam. The objectives of this study are to further explore the species diversity of Meruliaceae especially the corticioid group in subtropical and tropical areas, resolve taxonomic and phylogenetic problems of select taxa, determine the validity, circumscription, and relationship of select genera and contribute to the well-supported and robust phylogeny of the Meruliaceae. The phylogenetic analyses were carried out on two separate datasets of the ITS-LSU and five gene (ITS-nrLSU-*rpb1-rpb2-tef1*) sequences. Moreover, to understand the evolution history and to evaluate the generic delimitations, we estimated the divergence times of the family for the first time.

## 2. Materials and methods

### 2.1. Specimen collection and morphological studies

Field trips for specimen collection in various nature reserves and forest parks in China, Malaysia, Sri Lanka, and Vietnam were carried out by the authors. Voucher specimens are deposited at the herbaria of Beijing Forestry University, Beijing, China (BJFC), and the Center for Forest Mycology Research, Madison, Wisconsin, USA (CFMR). Herbarium code designations follow Index Herbarium (http://sweetgum.nybg.org/science/ih/). The methods of microscopic examinations and the abbreviations used in this paper follow Li et al. (2023).

### 2.2. DNA extraction and sequencing

The total genomic DNA was extracted from dried specimens by Kit DN14, a CTAB plant genomic DNA extraction (Aidlab Biotechnologies Co., Ltd., Beijing, China). The ITS, nrLSU, *rpb1*, *rpb2*, and *tef1-α* sequences were amplified with different primer pairs. The primer pairs and PCR (the polymerase chain reaction) cycling procedures for the five gene regions followed as given by Chen et al. (2021). The PCR products were purified and sequenced at the Beijing Genomics Institute (BGI), China. All newly generated sequences were deposited in GenBank (https://www.ncbi.nlm.nih.gov/).

### 2.3. Phylogenetic analyses

Two combined datasets, the ITS-nrLSU-*rpb1-rpb2-tef1* including 90 ITS, 88 nrLSU, 60 *rpb1*, 53 *rpb2*, and 50 *tef1-α* sequences from 93 samples representing 76 ingroup taxa and the ITS-nrLSU including 195 ITS and 167 nrLSU sequences from 202 samples representing 104 ingroup taxa, were compiled for phylogenetic analyses (Table 1). The taxa and sequences selections were consulted with Chen et al. (2021). *Phlebiopsis gigantea* (Fr.) Jülich and *Rhizochaete radicata* (Henn.) Gresl., Nakasone & Rajchenb. were selected as the outgroups. All the DNA sequences were aligned separately using MAFFT v.7 (http://mafft.cbrc.jp/alignment/server/) and optimised manually in BioEdit v.7.0.5.3 (Hall 1999). Mesquite v.3.5.1 (Maddison and Maddison 2018) was used to concatenate the separate alignments. The final alignments and the topologies were deposited in TreeBasee (http://treebase.org/treebase-web/home.html, submission ID: 31812).

**Table 1.** Taxa information and GenBank accession numbers of the sequences used in phylogenetic and molecular clock analyses.

Species	Specimen No.	Locality	GenBank Accession No.					Reference
			ITS	nrLSU	*rpb1*	*rpb2*	*tef1-α*	
*Agaricus campestris*	LAPAG370	Unknown	KM657927	KR006607	–	KT951556	KR006636	Zhou et al. (2016a)
***Allophlebia formosana***	**GC 1604–42**	**China**	**MZ637033**	**MZ637237**	**MZ748460**	**OK136056**	**MZ913648**	**Chen et al. (2021)**
***Allophlebia formosana***	**He 3805**	**China**	**PP549545ª**	**PP549577ª**	–	–	–	**Present study**
***Allophlebia formosana***	**He 4394**	**China**	**PP549546ª**	**PP549578ª**	–	–	–	**Present study**
*Allophlebia ludoviciana*	HHB-6564-Sp	USA	MZ637036	MZ637240	MZ748461	OK136057	MZ913661	Chen et al. (2021)
*Allophlebia ludoviciana*	HHB-8715-Sp	USA	KY948770	KY948846	KY948913	OK136058	MZ913662	Justo et al. (2017); Chen et al. (2021)
*Allophlebia ludoviciana*	He 5758	Sri Lanka	PP549547ª	–	–	PP566655ª	PP576330ª	Present study
*Amylocorticium cebennense*	HHB-2808	USA	GU187505	GU187561	GU187439	GU187770	GU187675	Binder et al. (2010)
*Antrodia serialis*	KHL 12010	Norway	JX109844	JX109844	–	JX109870	JX109898	Binder et al. (2013)
*Athelia arachnoidea*	CBS 418.72	Unknown	GU187504	GU187557	GU187436	GU187769	GU187672	Binder et al. (2010)
*Athelia epiphylla*	FP-100564	USA	GU187501	GU187558	GU187440	GU187771	GU187676	Binder et al. (2010)
***Aurantiopileus albidus***	**CIEFAP-117**	**Argentina**	**KY948739**	**KY948848**	**KY948925**	–	–	**Justo et al. (2017)**
***Aurantiopileus albidus***	**F32**	**Argentina**	**MT076170**	–	–	–	–	**Unpublished**
*Aurantiopileus mayaensis*	JV 1504/128	Costa Rica	KT156706	–	–	–	–	Unpublished
*Aurantiopileus mayaensis*	TJB10228	Belize	HM772140	HM772139	–	–	–	Ginns et al. (2010)
***Aurantiopileus semisupina***	**Cui 10222**	**China**	**KF845956**	**KF845949**	–	–	–	**Zhao and Cui (2014)**
***Aurantiopileus semisupina***	**Chen 3327**	**China**	**MZ636937**	**MZ637100**	**MZ748451**	**OK136061**	**MZ913700**	Chen et al. (2021)
*Aurantiporus alboaurantius*	Cui 2877	China	KF845954	KF845947				Zhao and Cui (2014)
*Aurantiporus alboaurantius*	Cui 4136	China	KF845955	KF845948	–	–	–	Zhao and Cui (2014)
*Aurantiporus croceus*	H6–27	Lithuania	MH571407	–	–	–	–	Unpublished
*Aurantiporus croceus*	BRNM 737561	Czech Republic	JQ821320	JQ821317	–	–	–	Dvořák et al. (2014)
*Aurantiporus mutans*	JV0309/83a	USA	MN318458	–	–	–	–	Unpublished
*Aurantiporus mutans*	JV0309/83b	USA	MN318459	–	–	–	–	Unpublished
*Aurantiporus orientalis*	Dai 23714	China	PP702380	PP623071	–	–	–	Zhang et al. (2024)
*Aurantiporus pseudoplacentus*	PRM 899297	USA	JN592497	JN592504	–	–	–	Vlasák et al. (2012)
*Aurantiporus pseudoplacentus*	Miettinen 18,997	USA	KY948744	KY948902	KY948926	–	–	Justo et al. (2017)
*Aurantiporus roseus*	Dai 13573	China	KJ698635	KJ698639	–	–	–	Zhao et al. (2015)
*Aurantiporus* sp.	Miettinen -16483	Malaysia	KY948745	KY948901	KY948927	–	–	Justo et al. (2017)
*Aurantiporus tropicus*	JV1707/5T	Costa Rica	MN318455	–	–	–	–	Unpublished
*Boletus edulis*	HMJAU4637	Unknown	JN563894	KF112455	KF112586	KF112704	KF112202	Feng et al. (2012)
*Bondarzewia montana*	AFTOL-ID 452	Unknown	DQ200923	DQ234539	DQ256049	AY218474	DQ059044	Matheny et al. (2007b)
*Bondarzewia* sp.	Yu 56	China	KT693203	KT693205	KX066158	KX066165	KX066148	Song et al. (2016)
*Byssomerulius corium*	FCUG 2701	Russia	MZ636931	GQ470630	MZ748415	OK136068	MZ913668	Wu et al. (2010); Chen et al. (2021)
*Ceriporiopsoides guidella*	HUBO 7659	Italy	FJ496687	FJ496722	–	–	–	Tomšovský et al. (2010)
*Ceriporiopsoides lagerheimii*	58240	Unknown	KX008365	KX081077	–	–	–	Zhao and Wu (2017)
*Cerrena unicolor*	FD-299	USA	KP135304	KP135209	KP134874	KP134968	–	Floudas and Hibbett (2015)
*Climacodon septentrionalis*	RLG-6890-Sp	USA	KP135344	–	–	–	–	Floudas and Hibbett (2015)
*Climacodon septentrionalis*	AFTOL-ID 767	Unknown	AY854082	AY684165	AY864872	AY780941	AY885151	Lutzoni et al. (2004)
*Crustodontia chrysocreas*	HHB-3946	USA	KP135357	–	–	–	–	Floudas and Hibbett (2015)
*Crustodontia chrysocreas*	HHB-6333-Sp	USA	KP135358	KP135263	KP134861	KP134908	–	Floudas and Hibbett (2015)
*Crustodontia nigrodontea*	CLZhao 2729	China	MT896823	MT896819	–	–	–	Huang et al. (2020a)
*Crustodontia nigrodontea*	CLZhao 2758	China	MT896824	–	–	–	–	Huang et al. (2020a)
*Crustodontia rhododendri*	CLZhao 6168	China	MW732400	MW724792	ON950240	–	ON892523	Zhao et al. (2023)
*Crustodontia rhododendri*	GC 1709–7	China	MZ636941	–	MZ748465	–	MZ913655	Chen et al. (2021)
*Crustodontia rhododendri*	KUC20121123–24	South Korea	KJ668482	–	–	–	–	Jang et al. (2016)
*Crustodontia rhododendri*	He 4617	China	PP549548ª	PP549579ª	–	–	–	Present study
*Crustodontia rhododendri*	He 6251	China	PP549549ª	PP549580ª	–	–	PP576333ª	Present study
*Crustodontia* sp.	CBS 125889	New Zealand	MH864087	MH875546	–	–	–	Vu et al. (2019)
*Crustodontia* sp.	HHB-17984	New Zealand	KP135359	KP135261	KP134860	KP134907	–	Floudas and Hibbett (2015)
*Crustodontia* sp.	HHB-18142	New Zealand	KP135360	–	–	–	–	Floudas and Hibbett (2015)
*Crustodontia* sp.	Wu 1809–169	China	MZ636942	MZ637104	–	–	–	Chen et al. (2021)
*Crustodontia* sp.	Wu 1809–201	China	MZ636943	MZ637105	–	–	–	Chen et al. (2021)
*Crustodontia taiwaniana*	GC 1703–88	China	MZ636944	MZ637106	MZ748466	OK136049	–	Chen et al. (2021)
*Crustodontia taiwaniana*	Wu 9310–21	China	MZ636945	MZ637107	–	–	–	Chen et al. (2021)
*Crustodontia tongxiniana*	CLZhao 2255	China	MT020773	MT020751	–	–	–	Huang and Zhao (2020)
*Crustodontia tongxiniana*	He 6168	China	PP549550ª	PP549581ª	–	–	PP576332ª	Present study
***Crustodontia vietnamensis***	**He 5224**	**Vietnam**	–	**PP549582ª**	–	–	–	**Present study**
***Crustodontia vietnamensis***	He 5232*	**Vietnam**	**PP549551ª**	**PP549583ª**	–	–	–	**Present study**
*Daedalea quercina*	FP-56429	USA	KY948809	KY948883	KY948989	–	–	Justo et al. (2017)
*Datronia mollis*	RLG-6304-Sp	USA	JN165002	JN164791	JN164818	JN164872	JN164901	Justo and Hibbett (2011)
*Echinodontium tinctorium*	AFTOL-ID 455	USA	AY854088	AF393056	AY864882	AY218482	AY885157	Unpublished
*Efibula tropica*	WEI 18–149	China	MZ636967	MZ637129	MZ748419	OK136079	MZ913681	Chen et al. (2021)
*Fomitiporia hartigii*	MUCL 53551	Belgium	JX093789	JX093833	–	JX093877	JX093746	Amalfi and Decock (2013)
*Fomitiporia mediterranea*	AFTOL-ID 688	USA	AY854080	AY684157	AY864870	AY803748	AY885149	Unpublished
*Fomitopsis pinicola*	AFTOL-770	Unknown	AY854083	AY684164	AY864874	AY786056	AY885152	Lutzoni et al. (2004)
*Geastrum recolligens*	OSC41996	Unknown	–	DQ218486	–	DQ219052	DQ219230	Hosaka et al. (2008)
*Geesterania carneola*	MCW 388/12	Brazil	KY174999	KY174999	–	KY175011	KY175013	Westphalen et al. (2018)
*Geesterania davidii*	MCW 396/12	Brazil	KY174998	KY174998	–	KY175012	KY175016	Westphalen et al. (2018)
*Gelatoporia subvermispora*	FD-354	USA	KP135312	KP135212	KP134879	–	–	Floudas and Hibbett (2015)
*Gloeophyllum sepiarium*	Wilcox-3BB	USA	HM536091	HM536061	–	HM536109	HM536110	Garcia-Sandoval et al. (2011)
*Gloeophyllum striatum*	ARIZ AN 027866	Unknown	HM536092	HM536063	–	HM640259	HM536111	Garcia-Sandoval et al. (2011)
*Gymnopilus picreus*	ZRL2015011	Unknown	LT716066	KY418882	KY418980	KY419027	KY419077	Zhao et al. (2017)
*Hermanssonia centrifuga*	CBS 125890	Sweden	MH864088	MH875547	–	–	–	Vu et al. (2019)
*Hermanssonia centrifuga*	HHB-9239-Sp	USA	KP135380	KP135262	KP134844	KP134974	MZ913721	Floudas and Hibbett (2015); Chen et al. (2021)
*Hermanssonia fimbriata*	Dai 23266	China	ON135436	ON135440	–	–	–	Liu et al. (2022)
*Hermanssonia fimbriata*	Dai 23305	China	ON135437	ON135441	–	–	–	Liu et al. (2022)
*Hydnophanerochaete odontoidea*	CLZhao 4036	China	MH784927	MH784937	–	–	–	Shen et al. (2018)
*Hydnophanerochaete odontoidea*	GC 1308–45	China	LC363486	LC363492	LC363497	LC387353	LC387373	Chen et al. (2018)
*Hydnophlebia alachuana*	L-11510-Sp	USA	KP135340	–	–	–	–	Floudas and Hibbett (2015)
*Hydnophlebia alachuana*	FP-103881-Sp	USA	KP135341	KP135201	KP134845	KP134896	–	Floudas and Hibbett (2015)
*Hydnophlebia aurantia*	WEI 18–623	China	MZ636982	MZ637143	MZ748459	OK136066	MZ913719	Chen et al. (2021)
*Hydnophlebia aurantia*	WEI 18–658	China	MZ636983	MZ637144	–	–	–	Chen et al. (2021)
*Hydnophlebia canariensis*	MA-Fungi 86622	Spain	KF483012	KF528103	–	–	–	Telleria et al. (2017)
*Hydnophlebia crocata*	Wu 1708–68	China	MZ636984	MZ637145	–	–	–	Chen et al. (2021)
*Hydnophlebia crocata*	Wu 1708–70	China	MZ636985	MZ637146	–	–	–	Chen et al. (2021)
*Hydnophlebia chrysorhiza*	HHB-18767	USA	KP135337	–	–	–	–	Floudas and Hibbett (2015)
*Hydnophlebia chrysorhiza*	FD-282	USA	KP135338	KP135217	KP134848	KP134897	–	Floudas and Hibbett (2015)
*Hydnophlebia gorgonea*	MA-Fungi 86659	Cape Verde	KF483049	KF528140	–	–	–	Telleria et al. (2017)
*Hydnophlebia omnivora*	KKN-112-Sp	USA	KP135334	KP135216	KP134846	–	–	Floudas and Hibbett (2015)
*Hydnophlebia omnivora*	ME-497	USA	KP135332	KP135218	KP134847	–	–	Floudas and Hibbett (2015)
*Hydnophlebia sinensis*	He 1924	China	MK860723	MK860733	–	–	–	Liu et al. (2020)
*Hydnophlebia sinensis*	He 5680	China	–	MK860734	–	–	PP576327ª	Liu et al. (2020); Present study
*Hydnophlebia subchrysorhiza*	He 2325	China	MK860718	MK860727	–	–	–	Liu et al. (2020)
*Hydnophlebia subchrysorhiza*	He 3886	China	MK860717	MK860726	–	–	–	Liu et al. (2020)
*Hyphoderma mutatum*	HHB-15479-Sp	USA	KP135296	KP135221	KP134870	KP134967	–	Floudas and Hibbett (2015)
*Hyphoderma setigerum*	FD-312	USA	KP135297	KP135222	KP134871	–	–	Floudas and Hibbett (2015)
*Irpex flavus*	Wu 0705–1	China	MZ636988	MZ637149	MZ748432	OK136087	MZ913683	Chen et al. (2021)
*Jaapia argillacea*	CBS 252.74	Netherlands	GU187524	GU187581	GU187463	GU187788	GU187711	Binder et al. (2010)
*Lactarius deceptivus*	AFTOL-ID 682	USA	AY854089	AY631899	AY864883	AY803749	AY885158	Unpublished
*Lepiota cristata*	ZRL20151133	Unknown	LT716026	KY418841	KY418963	KY418992	KY419048	Zhao et al. (2017)
*Leptosporomyces raunkiaeri*	HHB-7628	USA	GU187528	GU187588	GU187471	GU187791	GU187719	Binder et al. (2010)
“*Lilaceophlebia*” *subserialis*	FCUG 1434	Norway	–	AF141631	–	–	–	Unpublished
*Lopharia cinerascens*	FP-105043-Sp	USA	JN165019	JN164813	JN164840	JN164874	–	Justo and Hibbett (2011)
***Luteochaete odontoidea***	**He 4259**	**China**	–	**PP549584ª**	–	–	–	**Present study**
***Luteochaete odontoidea***	He 5815*	**Sri Lanka**	**PP549552ª**	**PP549585ª**	–	**PP566656ª**	**PP576331ª**	**Present study**
*Luteochaete* sp.	RLG-13514-Sp	USA	KP135363	–	–	–	–	Floudas and Hibbett (2015)
*Luteochaete* sp.	FP-110129-Sp	USA	KP135362	KP135274	KP134850	KP134898	–	Floudas and Hibbett (2015)
*Luteochaete subglobosa*	CLZhao 3475	China	MK881897	MK881787	–	–	–	Huang et al. (2020a)
*Luteochaete subglobosa*	GC 1605–4	China	MZ636995	MZ637156	MZ748455	OK136053	MZ913645	Chen et al. (2021)
*Luteoporia albocitrina*	Dai 19507	Sri Lanka	MT872218	MT872216	–	–	–	Liu and Yuan (2020)
*Luteoporia albocitrina*	Dai 19622	Sri Lanka	MT872219	MT872217	–	–	–	Liu and Yuan (2020)
*Luteoporia albomarginata*	Dai 15229	China	KU598873	KU598878	–	–	–	Wu et al. (2016)
*Luteoporia albomarginata*	GC 1702–1	China	LC379003	LC379155	LC379160	LC387358	LC387377	Chen et al. (2018)
*Luteoporia* cf. *lutea*	GC 1409–1	China	MZ636998	MZ637158	MZ748467	OK136050	MZ913656	Chen et al. (2021)
*Luteoporia* cf. *lutea*	He 5952	China	PP549554ª	PP549587ª	–	–	–	Present study
*Luteoporia straminea*	CLZhao 18947	China	MW732407	MW724799	–	–	–	Zhao et al. (2023)
*Luteoporia straminea*	He 5904	China	PP549553ª	PP549586ª	–	–	–	Present study
***Meruliella hainanensis***	He 3896*	**China**	**PP549555ª**	**PP549588ª**	–	–	–	**Present study**
***Meruliella lindtneri***	**GB-1027**	**Unknown**	**AB210076**	–	–	–	–	**Kamei et al. (2005)**
***Meruliella lindtneri***	**GB-501**	**Norway**	**KY948772**	**KY948847**	**KY948923**	–	–	**Justo et al. (2017)**
***Merulius croceum***	**HHB-1993-Sp**	**USA**	**KY948778**	**KY948853**	**KY948921**	–	–	**Justo et al. (2017)**
*Merulius fuscotuberculatus*	CLZhao 10227	China	MT020759	MT020737	–	–	–	Huang and Zhao (2020)
*Merulius fuscotuberculatus*	CLZhao 10239	China	MT020760	MT020738	–	–	–	Huang and Zhao (2020)
***Merulius leptospermi***	**Paulus 4122**	**New Zealand**	**HQ153413**	–	–	–	–	**Ghobad-Nejhad and Hallenberg (2012)**
*Merulius nantahaliensis*	HHB-2816-Sp	USA	KY948777	KY948852	KY948920	OK136063	MZ913701	Justo et al. (2017); Chen et al. (2021)
***Merulius pinicola***	He 5267*	**Vietnam**	**PP549556ª**	–	–	–	–	**Present study**
***Merulius pinicola***	**He 5273**	**Vietnam**	**PP549557ª**	–	–	–	–	**Present study**
***Merulius serialis***	**FCUG2868**	**USA**	**HQ153429**	–	–	–	–	**Ghobad-Nejhad and Hallenberg (2012)**
***Merulius serialis***	**UC2023146**	**USA**	**KP814195**	–	–	–	–	Rosenthal et al. (2017)
*Merulius sinensis*	CLZhao 2562	China	MW732401	MW724793	–	–	–	Zhao et al. (2023)
*Merulius tomentopileatus*	CLZhao 9563	China	MT020765	MT020743	–	–	–	Huang and Zhao (2020)
*Merulius tomentopileatus*	GC 1602–67	China	MZ637040	MZ637244	MZ748457	OK136064	MZ913702	Chen et al. (2021)
*Merulius tomentopileatus*	He 4629	China	–	PP549589ª	–	–	–	Present study
*Merulius tomentopileatus*	He 5356	China	–	PP549590ª	–	–	–	Present study
*Merulius tremellosus*	FBCC278	Sweden	LN611126	LN611126	–	–	–	Kuuskeri et al. (2015)
*Merulius tremellosus*	Wu 1109–73	China	MZ637041	MZ637245	MZ748458	OK136065	MZ913703	Chen et al. (2021)
*Mycoacia aurea*	RLG-5075-Sp	USA	KY948759	MZ637161	KY948918	–	MZ913720	Justo et al. (2017); Chen et al. (2021)
*Mycoacia aurea*	Chen 3780	China	MZ637000	MZ637160	–	–	–	Chen et al. (2021)
***Mycoacia beijingensis***	**He 9275**	**China**	**PQ536124**	**PQ536125**	–	–	–	**Present study**
*“Mycoacia”* cf. *kurilensis*	WEI 18–312	China	MZ637001	MZ637162	MZ748453	OK136045	MZ913722	Chen et al. (2021)
*“Mycoacia”* cf. *kurilensis*	WEI 18–324	China	MZ637002	MZ637163	–	–	–	Chen et al. (2021)
*Mycoacia fuscoatra*	GC 1705–1	China	MZ637004	MZ637165	MZ748447	OK136043	MZ913652	Chen et al. (2021)
*Mycoacia fuscoatra*	HHB-10782-Sp	USA	KP135365	KP135265	–	–	–	Floudas and Hibbett (2015)
*Mycoacia gilvescens*	Yuan 2752	China	KF845953	KF845946	–	–	–	Zhao and Cui (2014)
*Mycoacia gilvescens*	Chen 3340	China	MZ636936	MZ637099	MZ748446	OK136039	MZ913651	Chen et al. (2021)
*Mycoacia gilvescens*	BRNM 709970	Czech Republic	EU546104	FJ496721	–	–	–	Tomšovský and Ryvarden (2008)
*Mycoacia kunmingensis*	CLZhao 152	China	KX081072	KX081074	–	–	–	Zhao and Wu (2017)
*Mycoacia kunmingensis*	CLZhao 153	China	KX081073	KX081075	–	–	–	Zhao and Wu (2017)
*Mycoacia livida*	Miettinen 101	Russia	HQ153416	–	–	–	–	Ghobad-Nejhad and Hallenberg (2012)
*Mycoacia livida*	FBCC937	Finland	LN611122	LN611122	–	–	–	Kuuskeri et al. (2015)
*Mycoacia lividina*	HHB-9721-Sp	USA	KY948756	–	–	–	–	Justo et al. (2017)
*Mycoacia lividina*	HHB-4160-Sp	USA	KY948755	KY948849	KY948916	OK136041	MZ913659	Justo et al. (2017); Chen et al. (2021)
***Mycoacia neotuberculata***	**FCUG3157**	**Iran**	**HQ153427**	–	–	–	–	**Ghobad-Nejhad and Hallenberg (2012)**
***Mycoacia neotuberculata***	**Wu 1708–107**	**China**	**MZ637089**	**MZ637286**	**MZ748450**	**OK136042**	**MZ913660**	**Chen et al. (2021)**
*Mycoacia nothofagi*	HHB-4273-Sp	USA	KP135369	KP135266	–	–	–	Floudas and Hibbett (2015)
*Mycoacia nothofagi*	GC 1805–5	China	MZ637005	MZ637166	MZ748449	–	–	Chen et al. (2021)
*Mycoacia subfascicularis*	Wu 1004–11	China	MZ637008	–	MZ748448	OK136044	MZ913653	Chen et al. (2021)
*Mycoacia subfascicularis*	He 3487	China	PP549558ª	PP549591ª	–	–	–	Present study
*Mycoaciella bispora*	EL13_99	Estonia	–	AY586692	–	–	–	Larsson et al.(2004)
*Mycoaciella efibulata*	WEI 16–167	China	MZ637010	MZ637170	MZ748468	OK136051	MZ913657	Chen et al. (2021)
*Mycoaciella efibulata*	CLZhao 15876‡	China	MW732404	MW724796	ON892515	–	–	Zhao et al. (2023)
*Mycoaciella efibulata*	He 4549	China	PP549559ª	PP549592ª	–	–	–	Present study
*Mycoaciella efibulata*	He 5442	China	PP549560ª	PP549593ª	–	–	PP576326ª	Present study
*Odoria alborubescens*	BRNU 627479	Czech Republic	JQ821319	JQ821318	–	–	–	Dvořák et al. (2014)
*Odoria alborubescens*	BP106943	Hungary	MG097864	MG097867	MG213724	MG213723	–	Papp and Dima (2017)
*Obba rivulosa*	FP-135416-Sp	USA	KP135309	KP135208	KP134878	KP134962	–	Floudas and Hibbett (2015)
*Pappia fissilis*	814	Czech Republic	HQ728291	HQ729001	–	–	–	Tomšovský (2012)
*Pappia fissilis*	BRNM 699803	Czech Republic	HQ728292	HQ729002	–	–	–	Tomšovský (2012)
*Phaeophlebiopsis himalayensis*	Chen 3143	China	MZ637013	MZ637174	MZ748359	OK135992	MZ913633	Chen et al. (2021)
*Phanerochaete sordida*	Wu 1109–55	China	MZ422829	MZ637213	MZ748389	OK136017	MZ913638	Chen et al. (2021)
*Phanerochaetella angustocystidiata*	Wu 9606–39	China	MZ637020	GQ470638	MZ748422	OK136082	MZ913687	Wu et al. (2010); Chen et al. (2021)
*Phlebia acerina*	FBCC345	Russia	LN611083	LN611083	–	–	–	Kuuskeri et al. (2015)
*Phlebia acerina*	GC 1708–40	China	MZ637030	MZ637234	MZ748454	OK136062	MZ913698	Chen et al. (2021)
*Phlebia* aff. *subochracea*	FBCC295	Sweden	LN611116	LN611116	–	–	–	Kuuskeri et al. (2015)
*Phlebia* aff. *subochracea*	HHB-8494-Sp	USA	KY948768	KY948845	KY948912	OK136060	–	Justo et al. (2017); Chen et al. (2021)
“*Phlebia*” *coccineofulva*	OMC1242	USA	KY948765	–	–	–	–	Justo et al. (2017)
“*Phlebia*” *coccineofulva*	HHB-11466-Sp	USA	KY948766	KY948851	KY948915	OK136055	MZ913710	Justo et al. (2017); Chen et al. (2021)
*Phlebia floridensis*	FP-102562-T	USA	KP135386	–	–	–	–	Floudas and Hibbett (2015)
*Phlebia floridensis*	HHB-9905-Sp	USA	KP135383	KP135264	KP134863	KP134899	–	Floudas and Hibbett (2015)
*Phlebia niveomarginata*	CLZhao 18972	China	MW732409	MW724801	ON892518	ON925000	ON892529	Zhao et al. 9^2023^)
*Phlebia niveomarginata*	CLZhao 19089	China	MW732410	MW724802	–	–	–	Zhao et al. (2023)
*Phlebia poroides*	CLZhao 16121	China	MW732405	MW724797	ON892516	ON918560	–	Zhao et al. (2023)
*Phlebia poroides*	CLZhao 18421	China	MW732406	MW724798	ON892517	ON924999	ON892528	Zhao et al. (2023)
*Phlebia radiata*	FD-85	USA	KP135377	–	–	–	–	Floudas and Hibbett (2015)
*Phlebia radiata*	AFTOL-ID 484	Unknown	AY854087	AF287885	AY864881	AY218502	AY885156	Lutzoni et al. (2004)
*Phlebia rufa*	FBCC297	Sweden	LN611092	LN611092	–	–	–	Kuuskeri et al. (2015)
*Phlebia rufa*	HHB-14924	USA	KP135374	–	–	–	–	Floudas and Hibbett (2015)
*“Phlebia”* s.l. sp.	Chen 3678	China	MZ637037	MZ637242	–	–	–	Chen et al. (2021)
***Phlebicolorata austroasiana***	**Dai 17556**	**China**	**ON135439**	**ON135443**	–	–	–	**Liu et al. (2022)**
***Phlebicolorata austroasiana***	**He 5744**	**Sri Lanka**	**PP549561ª**	**PP549594ª**	–	–	**PP576329ª**	**Present study**
*Phlebicolorata brevispora*	FBCC1463	USA	LN611135	LN611135	–	–	–	Kuuskeri et al. (2015)
*Phlebicolorata brevispora*	HHB-7024-Sp	USA	MZ637032	MZ637236	MZ748452	OK136046	MZ913667	Chen et al. (2021)
*Phlebiodontia acanthocystis*	FP150571	USA	KY948767	KY948844	KY948914	–	–	Justo et al. (2017)
*Phlebiodontia acanthocystis*	GC 1703–30	China	LC387338	LC387343	–	LC387366	LC387384	Chen et al. (2018)
***Phlebiodontia caspica***	**FCUG3159**	**Iran**	**HQ153410**	–	–	–	–	**Ghobad-Nejhad and Hallenberg (2012)**
***Phlebiodontia fissurata***	**CLZhao 2900**	**China**	**MW732402**	**MW724794**	**ON892527**	**ON892536**	**ON968926**	**Zhao et al. (2023)**
*Phlebiodontia rajchenbergii*	MCW626	Brazil	OP265191	OP265189	–	–	OP271829	Motato-Vásquez et al. (2022)
*Phlebiodontia rajchenbergii*	MCW636	Brazil	OP265192	OP265190	–	–	OP271830	Motato-Vásquez et al. (2022)
*Phlebiodontia subochracea*	KGN 162/95	Sweden	EU118656	EU118656	–	–	–	Larsson (2007)
*Phlebiodontia subochracea*	FCUG 1161	Denmark	AF141630	–	–	–	–	Parmasto and Hallenberg (2000)
*Phlebiopsis gigantea*	FCUG 1417	Norway	MZ637051	AF141634	MZ748370	OK135996	MZ913623	Parmasto and Hallenberg (2000); Chen et al. (2021)
*Phlebiporia bubalina*	Dai 13168	China	KC782526	KC782528	–	–	–	Chen and Cui (2014)
*Phlebiporia bubalina*	Dai 15179	China	KY131843	KY131902	–	–	–	Wu et al. (2017)
***Phlebiporia crystallifera***	He 20120720–8*	**China**	**PP549562ª**	**PP549595ª**	–	–	–	**Present study**
***Phlebiporia crystallifera***	**He 5227**	**Vietnam**	**PP549563ª**	**PP549596ª**	–	–	–	**Present study**
***Phlebiporia odontoidea***	He 4903*	**China**	**PP549564ª**	**PP549597ª**	–	–	–	**Present study**
***Phlebiporia odontoidea***	**Wu 1210–7**	**China**	**MZ637061**	–	**MZ748390**	**OK136052**	**MZ913647**	**Chen et al. (2021)**
*Podoserpula ailaoshanensis*	ZJL2015015	China	KU324484	KU324487	–	–	KU324494	Zhou et al. (2016b)
*Porodaedalea chinensis*	Cui 10252	China	KX673606	MH152358	–	MH101479	MG585301	Dai et al. (2017)
***Porophlebia fimbriata***	**Dai 11672**	**China**	**KJ698633**	**KJ698637**	–	–	–	**Zhao et al. (2015)**
***Porophlebia fimbriata***	**Cui 1671**	**China**	**KJ698634**	**KJ698638**	–	–	–	**Zhao et al. (2015)**
*Pseudophlebia setulosa*	PH5105	Unknown	GU461313	GU461313	–	–	–	Moreno et al. (2011)
*Pseudophlebia setulosa*	HHB-6891-Sp	USA	KP135382	KP135267	KP134864	KP134901	MZ913650	Floudas and Hibbett (2015); Chen et al. (2021)
***Pseudophlebia vesiculosa***	He 5730*	**Sri Lanka**	**PP549565ª**	**PP549598ª**	–	–	**PP576328ª**	**Present study**
***Pseudophlebia vesiculosa***	**He 6403**	**Malaysia**	–	**PP549599ª**		**PP576325ª**	**PP576334ª**	**Present study**
*Pyrenogaster pityophilus*	OSC59743	Unknown	–	DQ218519	–	DQ219057	DQ219232	Hosaka et al. (2008)
*Rhizochaete radicata*	FD-123	USA	KP135407	KP135279	KP134816	KP134937	–	Floudas and Hibbett (2015)
*Russula emeticicolor*	FH12253	Germany	KT934011	KT933872	KT957382	KT933943	–	Looney et al. (2016)
*Sarcodontia amplissima*	FP 104176	USA	MZ322829	MZ322839	–	–	–	Nakasone et al. (2021)
*Sarcodontia amplissima*	He 2120	USA	MZ322830	MZ322840	–	–	–	Nakasone et al. (2021)
*Sarcodontia setosa*	BRNM 721609	Czech Republic	KX831470	KX831472	–	–	–	Tomšovský (2016)
*Sarcodontia setosa*	BRNM 761841	Czech Republic	KX831471	KX831473	–	–	–	Tomšovský (2016)
*Sarcodontia uda*	L-15019-Sp	USA	KY948763	–	–	–	–	Justo et al. (2017)
*Sarcodontia uda*	FP-101544-Sp	USA	KP135361	KP135232	KP134859	KP134909	MZ913649	Floudas and Hibbett (2015); Chen et al. (2021)
*Scopuloides allantoidea*	GC 1602–11	China	MZ637080	MZ637278	–	–	–	Chen et al. (2021)
*Scopuloides allantoidea*	WEI 16–060	China	MZ637081	MZ637279	MZ748463	OK136047	MZ913664	Chen et al. (2021)
*Scopuloides dimorpha*	FP-102935-Sp	USA	KP135353	KP135285	KP134855	KP134906	–	Floudas and Hibbett (2015)
*Scopuloides dimorpha*	He 3478	China	PP549566ª	PP549600ª	–	–	–	Present study
***Scopuloides ellipsoidea***	**He 3489**	**China**	**PP549567ª**	**PP549601ª**	–	–	–	**Present study**
***Scopuloides ellipsoidea***	**He 4681**	**China**	**PP549568ª**	**PP549602ª**	–	–	–	**Present study**
***Scopuloides ellipsoidea***	He 4760*	**China**	**PP549569ª**	**PP549603ª**	–	–	–	**Present study**
***Scopuloides ellipsoidea***	**He 5792**	**Sri Lanka**	**PP549570ª**	**PP549604ª**	–	–	–	**Present study**
***Scopuloides grandinioides***	He 6295*	**China**	**PP549571ª**	**PP549605ª**	–	**PP566657ª**	–	**Present study**
***Scopuloides grandinioides***	**RLG-5104-sp**	**USA**	**KP135351**	**KP135283**	**KP134852**	**KP134904**	–	**Floudas and Hibbett (2015)**
***Scopuloides grandinioides***	**HHB-11766-sp**	**USA**	**KP135348**	–	–	–	–	**Floudas and Hibbett (2015)**
*Scopuloides hydnoides*	FP-150473	USA	KP135355	KP135284	KP134854	–	–	Floudas and Hibbett (2015)
*Scopuloides hydnoides*	He 4507	China	PP549572ª	PP549606ª	–	–	–	Present study
*Scopuloides hydnoides*	He 4572	China	PP549573ª	PP549607ª	–	–	–	Present study
*Scopuloides rimosa*	HHB-15484-Sp	USA	KP135352	KP135281	KP134851	KP134902	MZ913665	Floudas and Hibbett (2015); Chen et al. (2021)
*Scopuloides rimosa*	He 3320	China	PP549574ª	PP549608ª	–	–	–	Present study
*Scopuloides rimosa*	He 3620	China	PP549575ª	PP549609ª	–	–	–	Present study
*Scopuloides* sp.	HHB-7042-Sp	USA	KP135350	KP135282	KP134853	KP134903	–	Floudas and Hibbett (2015)
*Serpula himantioides*	MUCL:30528	Belgium	GU187545	GU187600	GU187480	GU187808	GU187748	Binder et al. (2010)
*Skeletocutis nivea*	ES2008–1	Sweden	JX109858	JX109858	–	JX109886	JX109915	Binder et al. (2013)
*Steccherinum ochraceum*	KHL 11902	Sweden	JQ031130	JQ031130	–	JX109865	JX109893	Binder et al. (2013)
*Suillus pictus*	AFTOL-ID 717	Unknown	AY854069	AY684154	AY858965	AY786066	AY883429	Unpublished
*Thelephora ganbajun*	ZRL20151295	Unknown	LT716082	KY418908	KY418987	KY419043	KY419093	Zhao et al. (2017)
*Tomentella* sp.	AFTOL-ID 1016	USA	DQ835998	DQ835997	–	DQ835999	–	Matheny et al. (2007a)
*Trametes versicolor*	FP-135156-Sp	USA	JN164919	JN164809	JN164825	JN164850	JN164878	Justo and Hibbett (2011)
*Tyromyces chioneus*	FD-4	USA	KP135311	KP135291	KP134891	KP134977	–	Floudas and Hibbett (2015)

New taxa are set in bold. Type specimens of new species are marked with an asterisk (*). Newly generated sequences are labelled with a superscript a (ª). The type specimen of Mycoaciella brunneospina is noted by a double dagger (‡).

Maximum parsimony (MP), maximum likelihood (ML) analyses, and Bayesian inference (BI) were carried out by using PAUP* v.4.0b10 (Swofford 2002), RAxML-NG (Kozlov et al. 2019), and MrBayes 3.2.6 (Ronquist et al. 2012), respectively. The methods and parameter setting of the phylogenetic analyses followed Li et al. (2023). jModeltest v.2.17 (Darriba et al. 2012) was used to estimate the best-fit substitution models for BI. Four Markov chains were run for 4,000,000 for the 5-gene dataset, and 8,000,000 for the 2-gene dataset until the split deviation frequency value was lower than 0.01. Trees were sampled every 100th generation. The 25% of the trees, which represented the burn-in phase of the analyses, were discarded, and the remaining trees were used to calculate posterior probabilities (BPP) in the majority rule consensus tree.

### 2.4. Divergence time estimation

Divergence times were estimated using BEAST v2.6.5 (Bouckaert et al. 2014) with the dataset of 5.8S-nrLSU-*rpb1-rpb2-tef1* sequences (Table 1). The rates of evolutionary changes at nuclear acids were estimated using ModelTest 3.7 (Posada and Crandall 1998), and the GTR+I+G was selected as the best-fit model. A BEAST XML file was generated using BEAUti v2. The selection and detailed set of fossil calibrations followed Wang et al. (2023). The resulting log file was confirmed with convergence of the chains using Tracer v1.6 (Rambaut et al. 2013; http://tree.bio.ed.ac.uk/software/tracer/). An ultrametric Maximum Clade Credibility (MCC) tree with ≥ 0.8 posterior probability was summarised using TreeAnnotator v2.6.5, discarding 10% of states as burn-in annotating clades. FigTree v1.4.4 (http://tree.bio.ed.ac.uk/software/figtree/) was used to visualise the resulting tree and to obtain the means and 95% highest posterior density (HPD) (Drummond and Rambaut 2007). A 95% HPD marks the shortest interval that contains 95% of the values sampled. The international chronostratigraphic chart follows Cohen et al. (2013); updated, http://www.stratigraphy.org/ICSchart/ChronostratChart2022-10.pdf.

## 3. Results

### 3.1. Phylogenetic analyses

**Figure 1.**
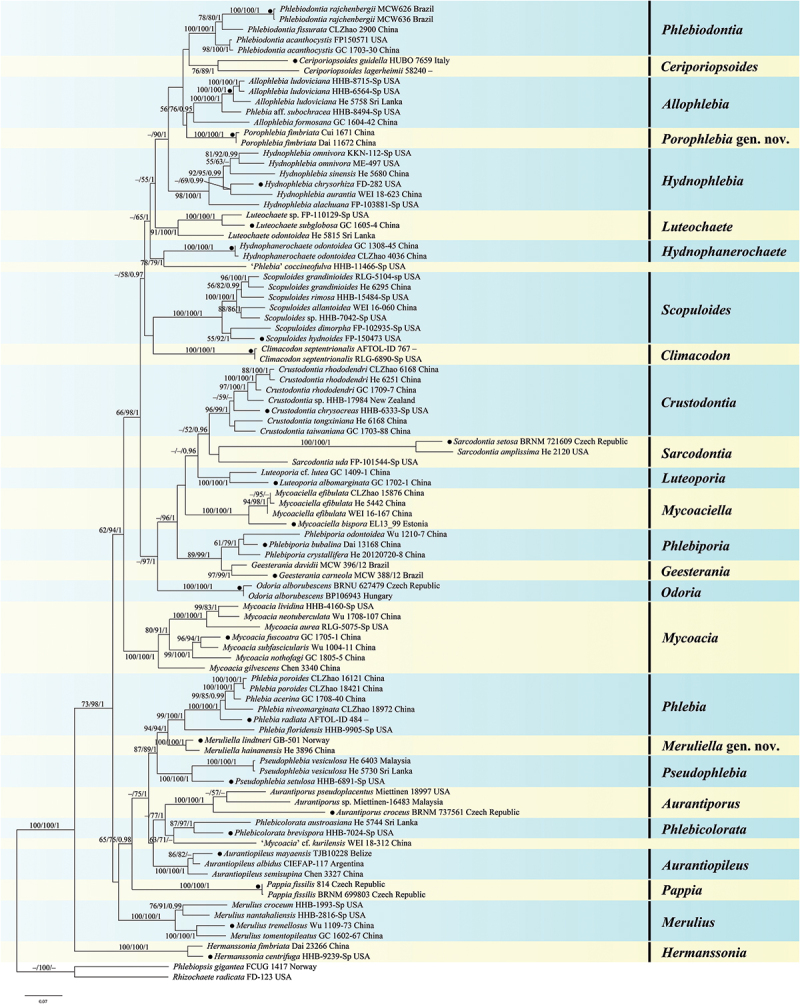
Phylogenetic tree of ML analysis from the ITS-nrLSU-*rpb1-rpb2-tef1* sequences of the Meruliaceae. Branches are labelled with parsimony bootstrap values (≥50%, first), likelihood bootstrap values (≥50%, second), and Bayesian posterior probabilities (≥0.95, third). Black circles (●) indicate the generic types.

The total aligned length of the concatenated five gene dataset was 5,498 characters with 1,961 variable characters and 512 singleton characters, whereas the concatenated ITS-nrLSU dataset had an aligned length of 2,489 characters with 838 variable characters and 205 singleton characters. The jModelTest suggested GTR+I+G as the best-fit model of nucleotide evolution for the two datasets. The average standard deviations of split frequencies of BI were 0.003918 and 0.006234 for the 5-gene and ITS-nrLSU datasets, respectively. The MP and BI analyses resulted in almost identical tree topologies with the ML analysis (Figures 1, 2).

**Figure 2.**
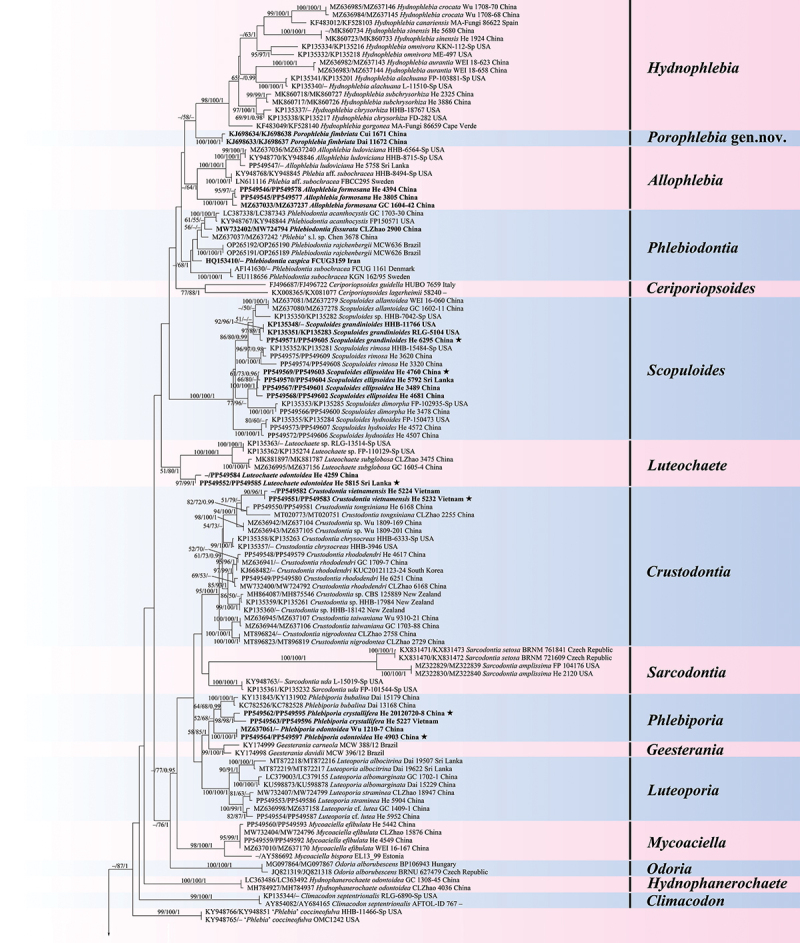
Phylogenetic tree of ML analysis from the ITS-nrLSU sequences of the Meruliaceae. Branches are labelled with parsimony bootstrap values (≥50%, first), likelihood bootstrap values (≥50%, second), and Bayesian posterior probabilities (≥0.95, third). New taxa are set in bold. Black stars (★) indicate the type specimens of new species.

**Figure 2.**
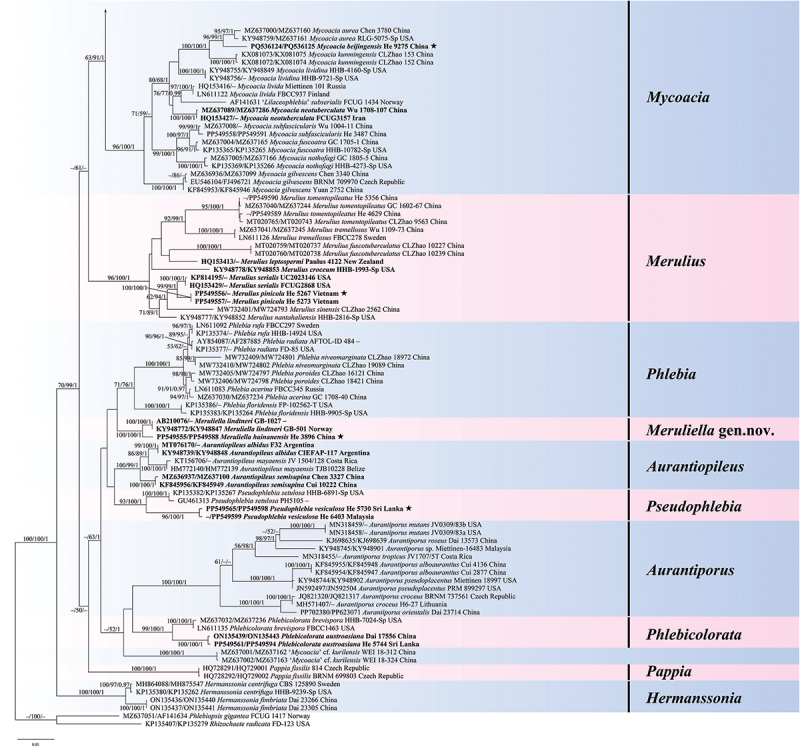
(Continued).

The topologies of the combined five gene and ITS-nrLSU trees are consistent in most lineages, except that *Phlebia*, *Meruliella*, and *Pseudophlebia* clustered together with 87/89/1 support values in the combined five gene tree, however *Aurantiopileus*, *Phlebia*, *Meruliella*, and *Pseudophlebia* clustered together with low support values in the ITS-nrLSU tree. In total, 28 supported lineages were recognised in the Meruliaceae including 24 established genera viz. *Allophlebia*, *Aurantiopileus*, *Aurantiporus*, *Ceriporiopsoides*, *Climacodon*, *Crustodontia*, *Geesterania*, *Hermanssonia*, *Hydnophanerochaete*, *Hydnophlebia*, *Luteochaete*, *Luteoporia*, *Merulius*, *Mycoacia*, *Mycoaciella*, *Odoria*, *Pappia*, *Phlebia*, *Phlebicolorata*, *Phlebiodontia*, *Phlebiporia*, *Pseudophlebia*, *Sarcodontia*, and *Scopuloides*. In addition, two new lineages are recognised and herein described as *Meruliella* gen. nov. and *Porophlebia* gen. nov., and two tentatively named lineages (“*Phlebia coccineofulva*” and “*Mycoacia* cf. *kurilensis*”). Three generic types, *Ceriporiopsis gilvescens* (Bres.) Domański, *Lilaceophlebia livida* (Pers.) Spirin & Zmitr. and *Mycoacia fuscoatra* (Fr.) Donk, were included in a lineage with other species of *Mycoacia* in the combined five gene tree with 100% MP and ML bootstrap values and 1.00 BYPP value and ITS-nrLSU tree with 96% MP and 100% ML bootstrap values and 1.00 BYPP value. The type species of *Noblesia*, *N. crocea* (Schwein.) Nakasone is nested within the *Merulius* lineage, and the type specimen of *Mycoaciella brunneospina* C.L. Zhao nested among samples of *M. efibulata* C.C. Chen & Sheng H. Wu. Ten distinct lineages, representing new species, were distributed among eight genera — *Crustodontia vietnamensis*, *Luteochaete odontoidea*, *Meruliella hainanensis*, *Merulius pinicola*, *Mycoacia beijingensis*, *Phlebiporia crystallifera*, *P. odontoidea, Pseudophlebia vesiculosa*, *Scopuloides ellipsoidea*, and *S. grandinioides*. Finally, the analyses indicated new generic placements for 12 species — *Allophlebia formosana*, *Aurantiopileus albidus*, *A*. *semisupina*, *Meruliella lindtneri*, *Merulius croceum*, *M. leptospermi*, *M*. *serialis*, *Mycoacia neotuberculata*, *Phlebicolorata austroasiana*, *Phlebiodontia caspica*, *P*. *fissurata*, and *Porophlebia fimbriata*.

### 3.2. Divergence time estimation

**Figure 3.**
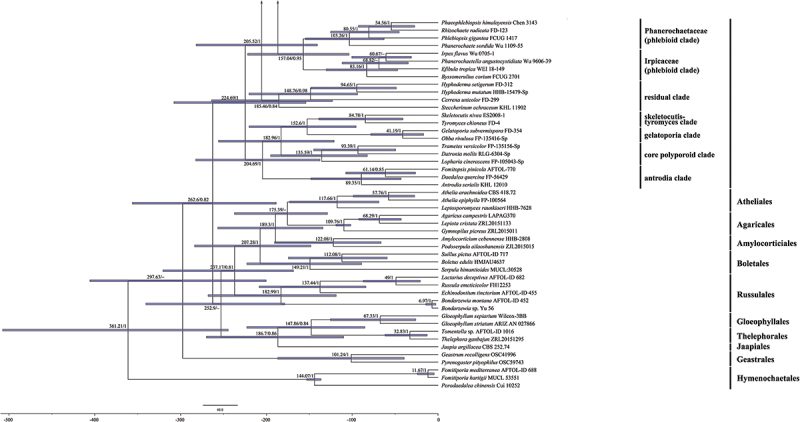
(Continued).

The combined dataset (5.8S-nrLSU-*rpb1-rpb2-tef1*) for the molecular clock analysis includes 133 fungal samples representing 117 taxa from 11 orders of Agaricomycetes, of which 85 samples are from 69 taxa of the Meruliaceae. The MCMC tree (Figure 3) shows that the ancestor of the phlebioid clade evolved during the late Triassic, with a mean stem age of 205.52 Mya with a 95% HPD of 140.64–281.91 Mya, whereas the Meruliaceae emerged with a mean stem age of 186.71 Mya with a 95% HPD of 127.53–259.9 Mya and a mean crown age of 169.46 Mya with a 95% HPD of 114.97–235.05 Mya, belonging to the Jurassic. The divergence times of estimated taxa of the Meruliaceae are summarised in Table 2. Among the genera of Meruliaceae, *Hermanssonia* (about 169.46 Mya) was the first to appear, and *Geesterania*/*Phlebiporia* (about 44.29 Mya) were the latest.
**Figure 3.**
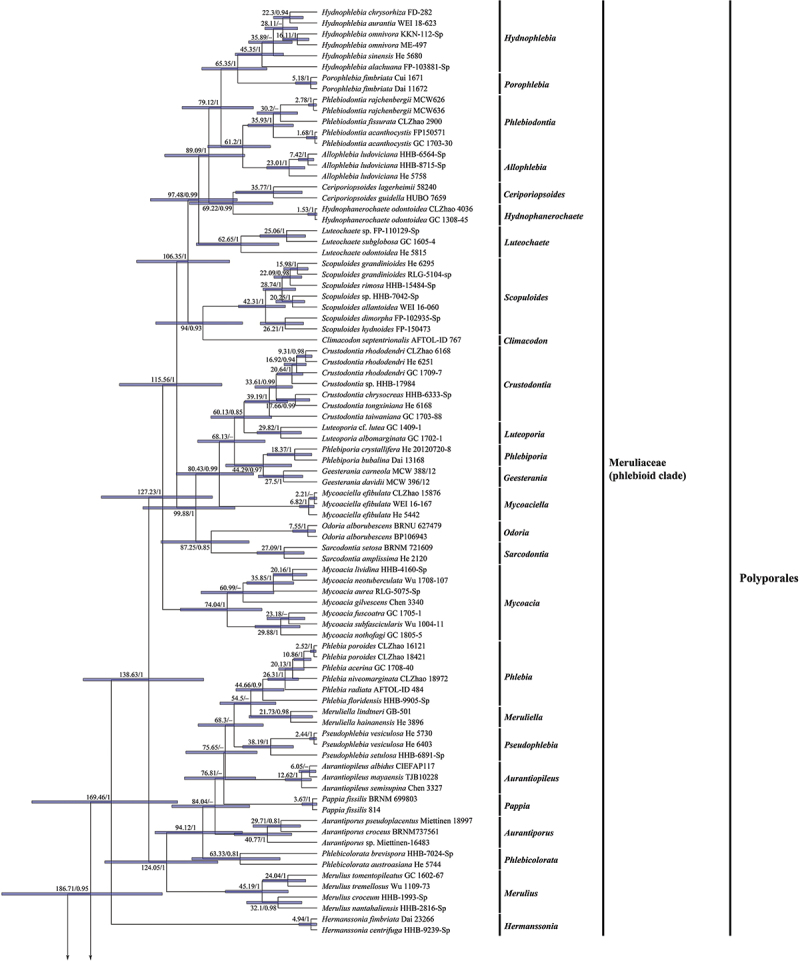
Divergence time estimation of the Meruliaceae within the Polyporales from Bayesian evolutionary analysis sampling tree based on 5.8S-nrLSU-*rpb1-rpb2-tef1* dataset. The mean ages (Mya) and posterior probabilities (≥0.80) of each node are annotated. The 95% highest posterior densities (HPD) of divergence time estimation are marked by horizontal bars.

**Table 2.** The divergence times of estimated taxa of the Meruliaceae.

Node	Mean of stem age (Mya)	95% HPD (Mya)	Posterior probabilities	Period
Meruliaceae	186.71	127.53–259.9	0.95	Early Jurassic
*Allophlebia*/*Phlebiodontia*	61.2	38.36–90.05	1	Paleocene
*Aurantiopileus*	75.65	49.36–108.14	0.54	Late Cretaceous
*Aurantiporus*	84.04	55.44–119.69	0.55	Late Cretaceous
*Ceriporiopsoides*/*Hydnophanerochaete*	69.22	36.43–105.28	0.99	Late Cretaceous
*Climacodon*/*Scopuloides*	94	61.37–132.95	0.93	Late Cretaceous
*Crustodontia*/*Luteoporia*	60.13	37.32–87.47	0.85	Paleocene
*Geesterania*/*Phlebiporia*	44.29	20.92–75.29	0.97	Eocene
*Hermanssonia*	169.46	114.97–235.05	1	Middle Jurassic
*Hydnophlebia*/*Porophlebia*	65.35	41.34–95	1	Paleocene
*Luteochaete*	97.48	65.73–137.3	0.99	Late Cretaceous
*Merulius*	124.05	81.75–174.89	1	Early Cretaceous
*Meruliella*/*Phlebia*	54.5	33.93–79.93	0.79	Eocene
*Mycoacia*	127.23	86.2–177.7	1	Early Cretaceous
*Mycoaciella*	80.43	52.3–116	0.99	Late Cretaceous
*Odoria*/*Sarcodontia*	87.25	55.97–128.58	0.85	Late Cretaceous
*Pappia*	76.81	50.53–109.26	0.33	Late Cretaceous
*Phlebicolorata*	94.12	61.47–133.47	1	Late Cretaceous
*Pseudophlebia*	68.3	44.31–97.99	0.73	Late Cretaceous

### 3.3. Taxonomy

**Meruliaceae** Rea, British Basidiomycetae: A handbook to the larger British fungi: 620, 1922.

*Type genus*: *Merulius* Fr., Systema Mycologicum 1: 326, 1821.

*Notes*: We recognise 26 genera, including two new genera, *Meruliella* gen. nov. and *Porophlebia* gen. nov., and two unnamed lineages as shown in Figures 1 and 2. These genera are discussed alphabetically below as well as the new species and new combinations proposed. New taxa are also described and illustrated.

***Allophlebia*** C.R.S. de Lira, Gibertoni & K.H. Larss., Mycological Progress 21 (5, no. 47): 5, 2022.

*Type species*: *Allophlebia ludoviciana* (Burt) C.R.S. de Lira & K.H. Larss., Mycological Progress 21 (5, no. 47): 5, 2022.

*Notes*: This monotypic genus was established to accommodate *Peniophora ludoviciana* Burt based on morphological characters and molecular data (Lira et al. 2022). The genus is characterised by having ceraceous basidiomes with smooth to slightly tuberculate hymenophores, clamped generative hyphae, two types of cystidia, and ellipsoid to narrowly ellipsoid basidiospores. *Crustodontia* is similar to *Allophlebia* by sharing yellow hymenophores that turning reddish or purplish in KOH, clamped generative hyphae, and ellipsoid to broadly ellipsoid basidiospores, but differs in having usually grandinioid-odontoid hymenophore, microbinding hyphae in some species, and cylindrical, subulate to ventricose gloeocystidia (Hjortstam and Ryvarden 2005; Chen et al. 2021; Zhao et al. 2023). *Allophlebia* is closely related to *Phebiodontia* and *Hydnophlebia* but differs from *Phebiodontia* in having golden yellow to deep orange basidiomes, lamprocystidia and allantoid basidiospores in some species (Lira et al. 2022), whereas *Hydnophlebia* is unique for its hydnoid or poroid hymenophores with rhizomorphic margin (Telleria et al. 2017; Liu et al. 2020; Chen et al. 2021).

***Allophlebia formosana*** (Sheng H. Wu) S. H. He, Yue Li & Nakasone, **comb. nov.**

MycoBank: MB 856581.

≡ *Phlebia formosana* Sheng H. Wu, Acta Botanica Fennica 142: 27, 1990.

≡ *Lilaceophlebia formosana* (Sheng H. Wu) Spirin & Zmitr., Novosti Sistematiki Nizshikh Rastenii 37: 179, 2004.

*Specimens examined*: China. Fujian Province, Jian’ou, Wanmulin Nature Reserve, on dead angiosperm branch, 19 August 2016, S.H. He, He 4546 (BJFC 023987, CFMR); Wuyishan, Wuyishan Nature Reserve, on fallen angiosperm trunk, 18 August 2016, S.H. He, He 4504 (BJFC 023945, CFMR); Guizhou Province, Libo County, Maolan Nature Reserve, on fallen angiosperm trunk, 16 June 2016, S.H. He, He 3805 (BJFC 022304); Jiangxi Province, Lianping County, Jiulianshan Nature Reserve, on fallen angiosperm trunk, 13 August 2016, S.H. He, He 4394 (BJFC 023835). U.S.A. Florida, Alachua County, University of Florida, Natural Area Teaching Laboratory, on rotting wood, 7 October 2017, A. Mujic, FLAS-F-61701 (CFMR); Putnam County, on wood, Ordway-Swisher Biological Station, east of Lake Rowan, 6 July 2017, D. Borland & B. Kaminsky, FLAS-F-61075 and F-61076 (CFMR).

*Notes*: Samples of *P. formosana* from mainland China and Taiwan of China formed a lineage sister to *A. ludoviciana* although their relationship was not well-supported (Figure 2). There are 57 base pair differences from a total of 569 between *A. ludoviciana* (HHB-6564-Sp) and *P. formosana* (He 4394). Both species develop yellowish, ceraceous basidiomes, clamped generative hyphae, and ellipsoid basidiospores, but the leptocystidia in the former are broader with obtuse apices (Nakasone et al. 1982; Wu 1990; Lira et al. 2022). Based on the present molecular evidence, we transfer *P. formosana* to *Allophlebia*. Earlier, Wu (1990) noted morphological similarities between *P. formosana* and *Phlebiodontia subochracea* (Bres.) Motato-Vásq. & Gugliotta whereas Spirin and Zmitrovich (2004) with the taxa in *Lilaceophlebia*. Note that the distribution of *P. formosana* is extended to North America for ITS sequences of the Florida samples cited above (MH212055, MH211728, MH211727) were 98%‒99% similar to those from China.

***Aurantiopileus*** Ginns, D.L. Lindner & T.J. Baroni, North American Fungi 5: 3, 2010.

*Type species*: *Aurantiopileus mayaensis* Ginns, D.L. Lindner & T.J. Baroni, North American Fungi 5: 4, 2010.

*Notes*: *Aurantiopileus* was introduced by Ginns et al. (2010) to accommodate *A. mayaensis* (generic type) from Belize and two Asian species transferred from *Gloeoporus*, *A. dolosus* (Corner) Ginns & D.L. Lindner, and *A. pendens* (Corner) Ginns & D.L. Lindner. The genus is characterised by having effused-reflexed or substipitate basidiomes with orange, greyish brown to reddish brown pore surfaces when dry, round to angular pores, monomitic hyphal system with clamped generative hyphae, clavate to fusoid cystidia when present, and ellipsoid to broadly ellipsoid basidiospores (Ginns et al. 2010). Although phylogenetically distantly related, *Odoria* resembles *Aurantiopileus* by having a similar hyphal system and basidiospores but is readily distinguished by its pileate basidiomes and the absence of cystidia (Papp and Dima 2017). Based on the molecular and morphological evidence, we recognise *Aurantiopileus*, *Pseudophlebia*, and *Meruliella* as closely related but distinct genera; the latter two genera are discussed more fully below. As shown in Figures 1 and 2 and by previous studies, *Aurantiopileus mayaensis*, *Aurantiporus albidus* and *Ceriporiopsis semisupina* formed a strongly supported lineage (Justo et al. 2017; Chen et al. 2021; Motato-Vásquez et al. 2022); thus, the transfers of *A. albidus* and *C*. *semisupina* to *Aurtantiopileus* are proposed below.

***Aurantiopileus albidus*** (Rajchenb. & Cwielong) S.H. He, Yue Li & Nakasone, **comb. nov.**

MycoBank: MB 856582.

≡ *Aurantiporus albidus* Rajchenb. & Cwielong, Mycotaxon 54: 428, 1995.

*Notes*: *Aurantiporus albidus* was originally described from Argentina (Rajchenberg 1995). Phylogenetically, *Aurantiporus albidus* is closely related to *Aurantiopileus mayaensis* (Figures 1, 2; Zhang et al. 2024). Morphologically, both species have poroid hymenophores, monomitic hyphal systems with clamped generative hyphae, and broadly ellipsoid basidiospores (Rajchenberg 1995; Ginns et al. 2010).

***Aurantiopileus semisupina*** (C.L. Zhao, B.K. Cui & Y.C. Dai) S.H. He, Yue Li & Nakasone, **comb. nov.**

MycoBank: MB 856583.

≡ *Ceriporiopsis semisupina* C.L. Zhao, B.K. Cui & Y.C. Dai, Phytotaxa 164: 23, 2014.

≡ *Pseudophlebia semisupina* (C.L. Zhao, B.K. Cui & Y.C. Dai) C.L. Zhao, J. Fungi 9 (3, no. 320): 33, 2023.

*Notes*: Zhao et al. (2023) transferred *C. semisupina* to *Pseudophlebia*, but their study did not include sequences of *A. mayaensis*. Both *C. semisupina* and *A. mayaensis* develop greyish brown to reddish brown pore surfaces when dry, round to angular pores, monomitic hyphal systems with clamped generative hyphae, and ellipsoid basidiospores but the former taxon differs in lacking embedded cystidia (Ginns et al. 2010; Zhao and Cui 2014).

***Aurantiporus*** Murrill, Bulletin of the Torrey Botanical Club 32 (9): 487, 1905.

*Type species*: *Aurantiporus croceus* (Pers.) Murrill, Mycologia 12 (1): 11, 1920.

*Notes*: The genus is characterised by having resupinate to effused-reflexed or pileate basidiomes with round to angular or irregular pores that are pale pink, pale brown, dark orange, brown to reddish brown when dry, a monomitic hyphal system with clamped generative hyphae, the absence of cystidia, and broadly ellipsoid to subglobose basidiospores. Seven species, *Aurantiporus alboaurantius* (C.L. Zhao, B.K. Cui & Y.C. Dai) Y.C. Dai et. al., *A. croceus*, *A. mutans* (Peck) Y.C. Dai, Xin Zhang, Vlasák, Ghobad-Nejhad & Yuan Yuan, *A. orientalis* Y.C. Dai, Xin Zhang, Ghobad-Nejhad & Yuan Yuan, *A. pseudoplacentus* (Vlasák & Ryvarden) J. Vlasák & P. Vampola, *A. roseus* (C.L. Zhao & Y.C. Dai) Zmitr., and *A. tropicus* (I. Lindblad & Ryvarden) Y.C. Dai, Xin Zhang, Vlasák, Ghobad-Nejhad & Yuan Yuan formed a strongly supported lineage in the ITS-LSU phylogenetic tree (Figure 2) that is consistent with results presented in Zhang et al. (2024). In contrast, Zhao et al. (2023) placed the four species, *A. alboaurantius*, *A. croceus*, *A. pseudoplacentus*, and *A. roseus*, in *Phlebicolorata*, typified by *Phlebia brevispora* Nakasone. Because the relationship between *Phlebicolorata* and *Aurantiporus* was not well-supported in Figures 1 and 2, we recognise these genera as distinct lineages. In addition, species in *Phlebicolorata* have smooth, tuberculate, or spinose hymenophores whereas *Aurantiporus* species are poroid.

***Ceriporiopsoides*** C.L. Zhao, J. Fungi 9 (3, no. 320): 12, 2023.

*Type species*: *Ceriporiopsoides guidella* (Bernicchia & Ryvarden) C.L. Zhao, J. Fungi 9 (3, no. 320): 12, 2023.

*Notes*: In addition to the type, this newly described genus includes one other species, *C. lagerheimii* (Læssøe & Ryvarden) C.L. Zhao. The genus is characterised by having resupinate, hard basidiomes with round to angular pores, a monomitic hyphal system with clamped generative hyphae, the absence of cystidia, and cylindrical basidiospores (Zhao et al. 2023). Although lacking in unique morphological features, it appears as a distinct lineage in Figures 1 and 2 and in other studies, also (Chen et al. 2021; Liu et al. 2022; Zhao et al. 2023). Because only ITS and LSU sequences are available, its proper placement in the Meruliaceae is unknown. In our analyses, *Ceriporiopsoides* formed a moderately supported lineage in the clade composed of *Hydnophlebia*, *Allophlebia*, and *Phlebiodontia* (Figures 1, 2).

***Climacodon*** P. Karst., Rev. Mycol. (Toulouse) 3 (9): 20, 1881.

*Type species*: *Climacodon septentrionalis* (Fr.) P. Karst., Rev. Mycol. (Toulouse) 3 (9): 20, 1881.

*Notes*: *Climacodon* sensu stricto is nested within the Meruliaceae, forming a strongly supported lineage (Figures 1, 2; Moreno et al. 2017). Morphologically, *Climacodon* is characterised by large, pileate, imbricate basidiomes with hydnoid hymenophores, a monomitic hyphal system with clamped generative hyphae, thick-walled cystidia, and ellipsoid basidiospores. This genus is unique in this family by having pileate basidiomes with hydnoid hymenophores.

***Crustodontia*** Hjortstam & Ryvarden, Synopsis Fungorum 20: 36, 2005.

*Type species*: *Crustodontia chrysocreas* (Berk. & M.A. Curtis) Hjortstam & Ryvarden, Synopsis Fungorum 20: 36, 2005.

*Notes*: *Crustodontia*, widely distributed in subtropical and tropical areas, is characterised by having ceraceous to subceraceous hymenophores that turn reddish or purplish in KOH, clamped generative hyphae, sometimes with microbinding hyphae, cylindrical, subulate to ventricose cystidia, and ellipsoid to broadly ellipsoid basidiospores (Hjortstam and Ryvarden 2005). This is a distinct lineage that is related to *Sarcodontia* (Figures 1, 2; Chen et al. 2021; Zhao et al. 2023). The *Crustodontia* clade contains eight lineages including five known species, a new species described below, and two unnamed taxa from New Zealand and China. *Luteoporia* resembles *Crustodontia* but differs in having poroid or odontoid hymenophores and lacking cystidia (Wu et al. 2016; Chen et al. 2021).

**Figure 4.**
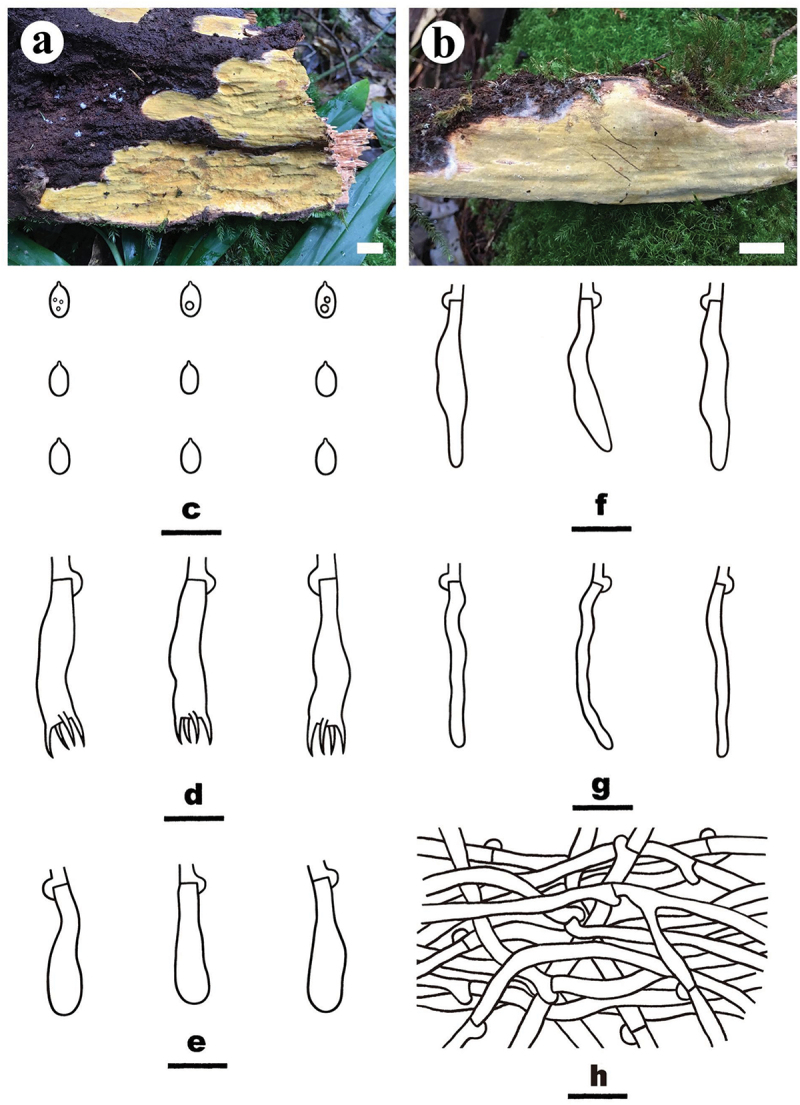
*Crustodontia vietnamensis* (drawn from the holotype, He 5232). (a, b) Basidiomes (a He 5232, b He 5224). (c) Basidiospores. (d) Basidia. (e) Basidioles. (f) Cystidia. (g) Hyphidia. (h) Hyphae from subiculum. Scale bars: a – b = 1 cm, c – h = 10 µm.

***Crustodontia vietnamensis*** S.H. He, Yue Li & Nakasone, **sp. nov**., Figure 4

MycoBank: MB 856585.

*Diagnosis*: The species is recognised by a smooth hymenophore with a greyish yellow to greyish orange hymenial surface that turning dark purple in KOH, the presence of subcylindrical to subfusiform cystidia, and ellipsoid basidiospores.

*Type*: Vietnam. Lam Dong Province, Bi Doup Nui Ba National Park, on fallen angiosperm trunk, 14 October 2017, S.H. He, He 5232 (holotype, BJFC 024750).

*Etymology*: *vietnamensis* (Lat.): refers to the type locality in Vietnam.

*Fruiting body*: Basidiomes annual, resupinate, widely effused, closely adnate, inseparable from substrate, ceraceous, first as small patches, later confluent up to 9 cm long, 3.5 cm wide, up to 80 µm thick in section. Hymenophore smooth, greyish yellow (4B5) to greyish orange [5B(5–6)], turning dark purple in KOH, not cracked; margin thinning out, adnate, indistinct, concolorous with hymenophore surface. Context greyish yellow (4B5).

*Microscopic structures*: Hyphal system monomitic; generative hyphae with clamp connections. Subiculum thin; hyphae colourless, thin- to slightly thick-walled, smooth, infrequently branched, moderately septate, densely interwoven, 2–3 µm in diam. Subhymenium with compact texture, yellowish resinous materials abundant; hyphae colourless, thin- to slightly thick-walled, smooth, infrequently branched, moderately septate, more or less vertical, interwoven, agglutinated, 2–2.5 µm in diam. Hyphidia sinuous, colourless, thin-walled, smooth, 22–27 × 1.5–2 µm. Cystidia subcylindrical to subfusiform, tapering towards the apex, colourless, thin-walled, smooth, 20–28 × 3.5–4.5 µm. Basidia clavate to subcylindrical, colourless, thin-walled, smooth, with a basal clamp connection and four sterigmata, 20–25 × 5–5.5 µm; basidioles similar to basidia but slightly smaller. Basidiospores ellipsoid, occasionally containing one or two oil-drops, colourless, thin-walled, smooth, IKI–, CB–, (4.2–) 4.5–5 (−5.2) × (2.8–) 3–3.5 (−3.8) µm, L = 4.8 µm, W = 3.1 µm, Q = 1.5–1.6 (*n* = 60/2).

*Additional specimen examined*: Vietnam. Lam Dong Province, Bi Doup Nui Ba National Park, on fallen angiosperm trunk, 14 October 2017, S.H. He, He 5224 (BJFC 024742).

*Notes*: *Crustodontia vietnamensis* is characterised by having greyish yellow to greyish orange, smooth hymenophores, subcylindrical to subfusiform cystidia, and ellipsoid basidiospores. Phylogenetically, *Crustodontia vietnamensis*, *C. tongxiniana* (C.L. Zhao) C.C. Chen & Sheng H. Wu and *Crustodontia* sp. (Wu 1809–169 and Wu 1809–201 from Guangxi Autonomous Region, China) formed a strongly supported lineage (Figure 2). *Crustodontia tongxiniana* can be distinguished from *C. vietnamensis* by the absence of cystidia (Huang and Zhao 2020; Chen et al. 2021). The ITS similarity between *C. vietnamensis* (holotype) and *C. tongxiniana* (CLZhao 2255, holotype) is 95.8% with 23 differences of total 554 base pairs, whereas there are 29 differences of the ITS sequences between *C. vietnamensis* (holotype) and *Crustodontia* sp. (Wu 1809–169).

***Geesterania*** Westph., Tomšovský & Rajchenb., Persoonia 41: 134, 2018.

*Type species*: *Geesterania carneola* (Bres.) Westph. & Rajchenb., Persoonia 41: 135, 2018.

*Notes*: The poroid genus *Geesterania* was created to accommodate *Poria carneola* Bres. and *G. davidii* Westphalen & Rajchenb (Westphalen et al. 2018). The genus is unique because of its subfleshy, soft, resupinate basidiomes that bruise reddish and when dried, round to angular pores, a dimitic hyphal system with clamped generative hyphae and skeletal hyphae, encrusted skeletocystidia, and ellipsoid to subcylindrical basidiospores. In Figures 1 and 2, *Geesterania* formed a distinct lineage and is sister to *Phlebiporia* that is easily distinguished by having a monomitic hyphal system with simple-septate, dextrinoid hyphae and lacking cystidia (Chen and Cui 2014).

***Hermanssonia*** Zmitr., Folia Cryptogamica Petropolitana 6: 100, 2018.

*Type species*: *Hermanssonia centrifuga* (P. Karst.) Zmitr., Folia Cryptogamica Petropolitana 6: 100, 2018.

*Notes*: Zmitrovich (2018) erected this genus for *Phlebia centrifuga* P. Karst. that consistently formed a basal lineage in the Meruliaceae in our analyses and previous work (Figures 1, 2; Justo et al. 2017; Chen et al. 2021; Liu et al. 2022; Zhao et al. 2023). Morphologically, *Hermanssonia* is characterised by ceraceous basidiomes with papillose to wrinkled hymenophores with strigose, fimbriate margins, clamped generative hyphae, the absence of cystidia, and cylindrical, ellipsoid to oblong ellipsoid basidiospores. *Hermanssonia fimbriata* Z.B. Liu & Y.C. Dai, from Xizang, China, was recently described by Liu et al. (2022).

***Hydnophanerochaete*** Sheng H. Wu & C.C. Chen, MycoKeys 39: 85, 2018.

*Type species*: *Hydnophanerochaete odontoidea* (Sheng H. Wu) Sheng H. Wu & C.C. Chen, MycoKeys 39: 86, 2018.

*Notes*: This monotypic genus was established to accommodate *Phanerochaete odontoidea* Sheng H. Wu, which has ceraceous basidiomes with odontoid to hydnoid hymenophores, simple-septate generative hyphae, absence of cystidia, and ellipsoid to cylindrical basidiospores (Chen et al. 2018). See Chen et al. (2018) for a detailed morphological comparison between *Hydnophanerochaete* and *Hydnophlebia*. *Luteoporia* is similar to *Hydnophanerochaete* also but differs in having clamped generative hyphae (Wu et al. 2016).

***Hydnophlebia*** Parmasto, Eesti NSV Teaduste Akadeemia Toimetised 16: 384, 1967.

*Type species*: *Hydnophlebia chrysorhiza* (Eaton) Parmasto, Eesti NSV Teaduste Akadeemia Toimetised 16: 384, 1967.

*Notes*: *Hydnophlebia* was segregated from *Phanerochaete* because of its bright yellow, hydnoid hymenophore, and rhizomorphs (Parmasto 1967). Researchers have described new species and transferred others so that Index Fungorum lists 14 names in the genus (Telleria et al. 2017; Liu et al. 2020; Chen et al. 2021; Zhao et al. 2023). Trees in Figure 2 differ from that in Zhao et al. (2023) for *H. acanthocystis* (Gilb. & Nakasone) C.L. Zhao, *H. caspica* (Hallenb.) C.L. Zhao and *Hydnophlebia fissurata* C.L. Zhao nested within *Phlebiodontia* and not *Hydnophlebia* (Motato-Vásquez et al. 2022); see further discussion under *Phlebiodontia* and *Allophlebia*. Additionally, *H. fimbriata* (C.L. Zhao & Y.C. Dai) C.L. Zhao formed a distinct lineage, labelled as *Porophlebia fimbriata* in Figures 1 and 2; see discussion below under *Porophlebia*.

***Luteochaete*** C.C. Chen & Sheng H. Wu, Fungal Diversity 111: 425, 2021.

*Type species*: *Luteochaete subglobosa* (Sheng H. Wu) C.C. Chen & Sheng H. Wu, Fungal Diversity 111: 425, 2021.

*Notes*: *Luteochaete* was recently introduced by Chen et al. (2021) to accommodate *Phanerochaete subglobosa* Sheng H. Wu and an undescribed species from the United States. It formed a monophyletic lineage that included an additional taxon from Sri Lanka (Figures 1, 2). Originally, *Luteochaete* was described with thick (up to 1,000 µm), effused basidiomes with a smooth hymenophore that turned slightly greenish yellow in KOH, simple-septate hyphae, lamprocystidia, and ellipsoid to subglobose basidiospores. The authors noted similarities to *Phaeophlebiopsis* Floudas & Hibbett and *Phlebiopsis* Jülich in the Phanerochaetaceae (Chen et al. 2021). With the addition of *L. odontoidea* described below, the genus circumscription must be emended to include basidiomes without a reaction to KOH, with odontoid hymenophores, clamped generative hyphae, hyphidia, and ellipsoid to subcylindrical basidiospores.

**Figure 5.**
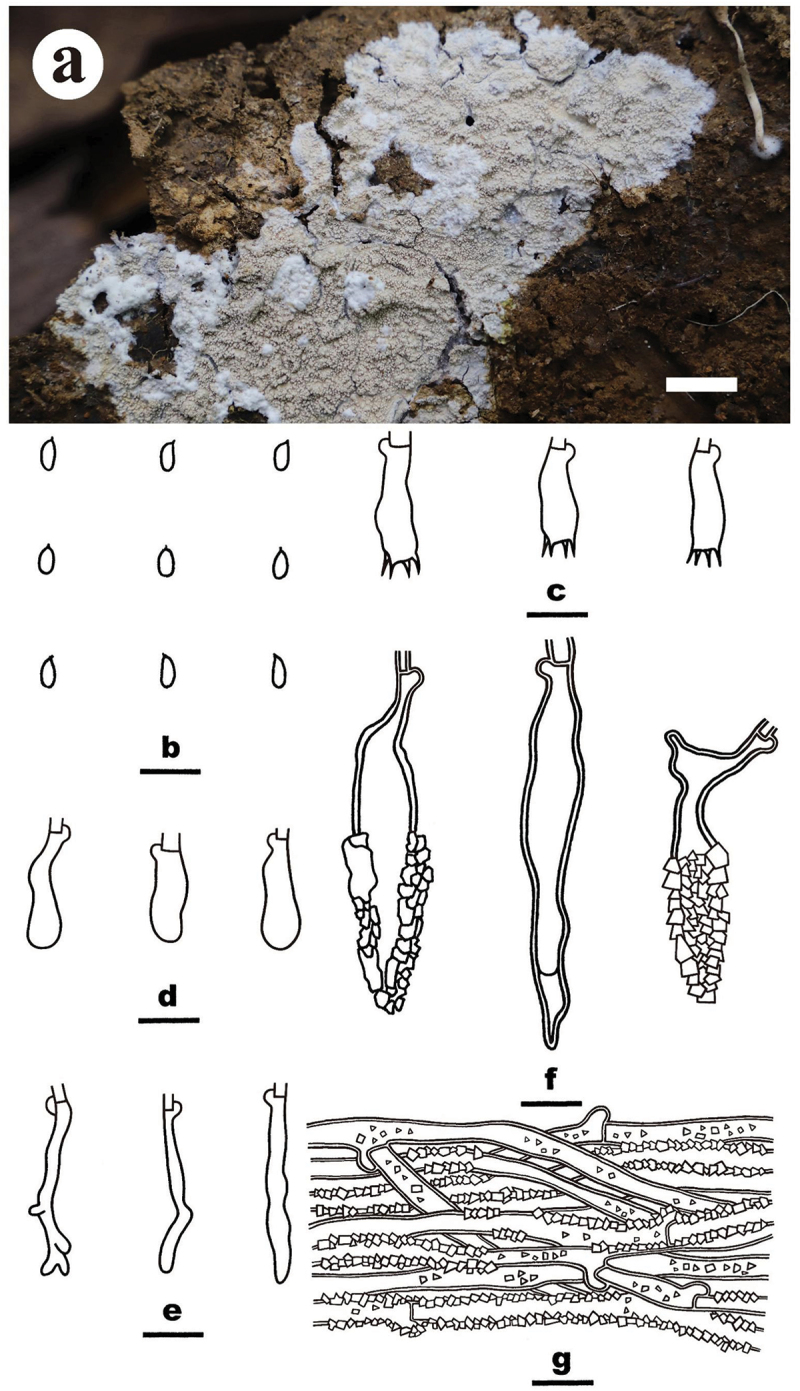
*Luteochaete odontoidea* (drawn from the holotype, He 5815). (a) Basidiomes (He 5815). (b) Basidiospores. (c) Basidia. (d) Basidioles. (e) Hyphidia. (f) Lamprocystidia. (g) Hyphae from subiculum. Scale bars: a = 1 cm, b – g = 10 µm.

***Luteochaete odontoidea*** S.H. He, Yue Li & Nakasone, **sp. nov**., Figure 5

MycoBank: MB 856586.

*Diagnosis*: The species is recognised by an odontoid hymenophore with a greyish orange hymenial surface and fimbriate margin, the presence of subfusiform lamprocystidia, and ellipsoid to subcylindrical basidiospores.

*Type*: Sri Lanka. Avissawella, Salgala Forest, on rotten angiosperm trunk, 3 March 2019, S.H. He, He 5815 (holotype, BJFC 030682).

*Etymology*: *odont*- (Greek): refers to the toothed hymenophore.

*Fruiting body*: Basidiomes annual, resupinate, widely effused, closely adnate, inseparable from substrate, coriaceous, first as small patches, later confluent up to 7.5 cm long, 6.5 cm wide, up to 300 µm thick in section (aculei excluded). Hymenophore odontoid, greyish orange [5B(4–5)], darkening after drying, unchanged in KOH, not cracked; margin thinning out, adnate, fimbriate, white. Aculei conical to cylindrical, usually separate, 2–4 per mm, up to 700 μm long. Context greyish yellow.

*Microscopic structures*: Hyphal system monomitic; generative hyphae with clamp connections. Subiculum distinct, agglutinated; hyphae colourless, slightly thick-walled, covered with abundant tiny crystalline granules, infrequently branched, moderately septate, more or less parallel to the substrate, 2–4 µm in diam. Subhymenium distinct, thickening; hyphae colourless, slightly thick-walled, smooth, infrequently branched, rarely septate, more or less vertical, interwoven, agglutinated, 2–3 µm in diam. Hyphidia sinuous, colourless, thin-walled, smooth, 20–26 × 2–3.5 µm. Lamprocystidia abundant, subfusiform, colourless, thick-walled, usually heavily encrusted in the upper part, 18–52 × 6–15 µm (with encrustation), projecting beyond the hymenium up to 40 µm, also embedded in subhymenium. Basidia subcylindrical, colourless, thin-walled, smooth, with a basal clamp connection and four sterigmata, 10–14 × 4–5.5 µm; basidioles similar to basidia but slightly smaller. Basidiospores ellipsoid to subcylindrical, with an apiculus, colourless, thin-walled, smooth, IKI–, CB–, (3.1–) 3.2–4 (−4.1) × (1.8–) 2–2.2 (−2.4) µm, L = 3.5 µm, W = 2.1 µm, Q = 1.6–1.8 (*n* = 60/2).

*Additional specimen examined*: China. Jiangxi Province, Yifeng County, Guanshan Nature Reserve, on fallen angiosperm trunk, 10 August 2016, S.H. He, He 4259 (BJFC 023701).

*Notes*: *Luteochaete odontoidea* is characterised by having odontoid hymenophores, clamped generative hyphae, hyphidia, subfusiform lamprocystidia, and small ellipsoid to subcylindrical basidiospores. *Luteochaete subglobosa* is easily distinguished from *L. odontoidea* for its smooth hymenophores, simple-septate generative hyphae, and larger basidiospores (5.3–6 × 3.3–3.8 µm; Wu 1990; Chen et al. 2021). The phylogenetic relationship between the two species was well-supported in the multi-gene tree and moderately supported in the ITS-LSU tree (Figures 1, 2).

***Luteoporia*** F. Wu, Jia J. Chen & S.H. He, Phytotaxa 263: 37, 2016.

*Type species*: *Luteoporia albomarginata* F. Wu, Jia J. Chen & S.H. He, Phytotaxa 263: 37, 2016.

*Notes*: *Luteoporia* formed a strongly supported distinct lineage in the Meruliaceae (Figures 1, 2). The original species in the genus were poroid (Wu et al. 2016; Liu and Yuan 2020), but later Chen et al. (2021) transferred the odontoid species, *Odontia lutea* G. Cunn. to the genus. *Luteoporia* is further characterised by hard, crustaceous basidiomes that turns red or pink in KOH, a monomitic hyphal system with clamped hyphae, fusoid cystidioles, and ellipsoid to cylindrical basidiospores (Chen et al. 2021).

However, it needs to be further studied whether the voucher samples NZFS2926 from New Zealand and GC 1409–1 from China represent *O*. *lutea*. Thus, we name the lineage of GC 1409–1 and He 5952 as *Luteoporia* cf. *lutea*. Zhao et al. (2023) described a new species, *Luteoporia straminea* C.L. Zhao, from southwestern China, that is phylogenetically closely related to *L*. cf. *lutea* but with some morphological differences including the lack of aculei composed of encrusted, fascicles and microbinding hyphae and the development of subulate, thick-walled cystidia and thin-walled basidiospores. There are 13 nucleotide differences of 549 total base pairs (2.4%) between the ITS sequences of *L. straminea* (CLZhao 18947, holotype) and *Luteoporia* cf. *lutea* (GC 1409–1). Based on available morphological and molecular data, we consider *L. straminea* a distinct taxon.

***Meruliella*** S.H. He, Yue Li & Nakasone, **gen. nov.**

MycoBank: MB 856587.

*Diagnosis*: The genus is recognised by a merulioid or alveolate to reticulate hymenophore with a light yellow, pale ochraceous, greyish orange to grey hymenial surface and fimbriate margin, the presence of conical, subclavate to subfusiform cystidia, and oblong-ellipsoid to subcylindrical basidiospores.

*Type species*: *Meruliella lindtneri* (Pilát) S.H. He, Yue Li & Nakasone.

*Etymology*: *Merulius* + *ella* (Lat.): a diminutive form of *Merulius*, indicating a similarity to the genus in morphology.

Basidiomes annual, resupinate, effused, adnate, ceraceous to subgelatinous when fresh, becoming hard and brittle upon drying. Hymenophore merulioid or alveolate to reticulate, light yellow, pale ochraceous, greyish orange to grey, unchanged in KOH, not cracked; margin thinning out, fimbriate. Hyphal system monomitic, generative hyphae with clamp connections; subicular hyphae embedded in a gelatinous matrix. Cystidia conical, subclavate to subfusiform, thin- to thick-walled, smooth, or encrusted with crystals. Basidia narrowly clavate to subcylindrical, with four sterigmata. Basidiospores oblong-ellipsoid to subcylindrical, colourless, thin-walled, smooth, IKI–, CB–. Associated with a white rot of wood.

*Notes*: In the previous analyses of ITS-LSU sequences (Chen et al. 2021; Lira et al. 2022), *Meruliella* (as *Phlebia lindtneri*) formed a distinct lineage sister to *Pseudophlebia* (as *Phlebia setulosa*) but distantly related to *Phlebia* s.s. In multi-gene trees and our ITS-LSU tree (Figures 1, 2; Justo et al. 2017; Chen et al. 2021), however, these genera clustered together with moderate support. We choose to recognise these taxa as separate genera and establish the new genus *Meruliella* for *P*. *lindtneri* and the new species described below. Morphologically, *Phlebia* s.s. and *Merulius* differ from *Meruliella* by having less gelatinous basidiomes and the absence of thick-walled cystidia. *Pseudophlebia* differs from *Meruliella* by developing coriaceous basidiomes with hydnoid hymenophores whereas *Aurantiopileus* produces pores from basidiomes with an effused-reflexed to pileate habit (see discussion above). Zhao et al. (2023) proposed a different solution by creating *Pseudophlebia* to accommodate *P*. *lindtneri*, *P. setulosa, Aurantiopileus mayaensis*, and *Ceriporiopsis semisupina* based on a weakly-supported lineage and a few morphological features; *Pseudophlebia* is discussed below.

**Figure 6.**
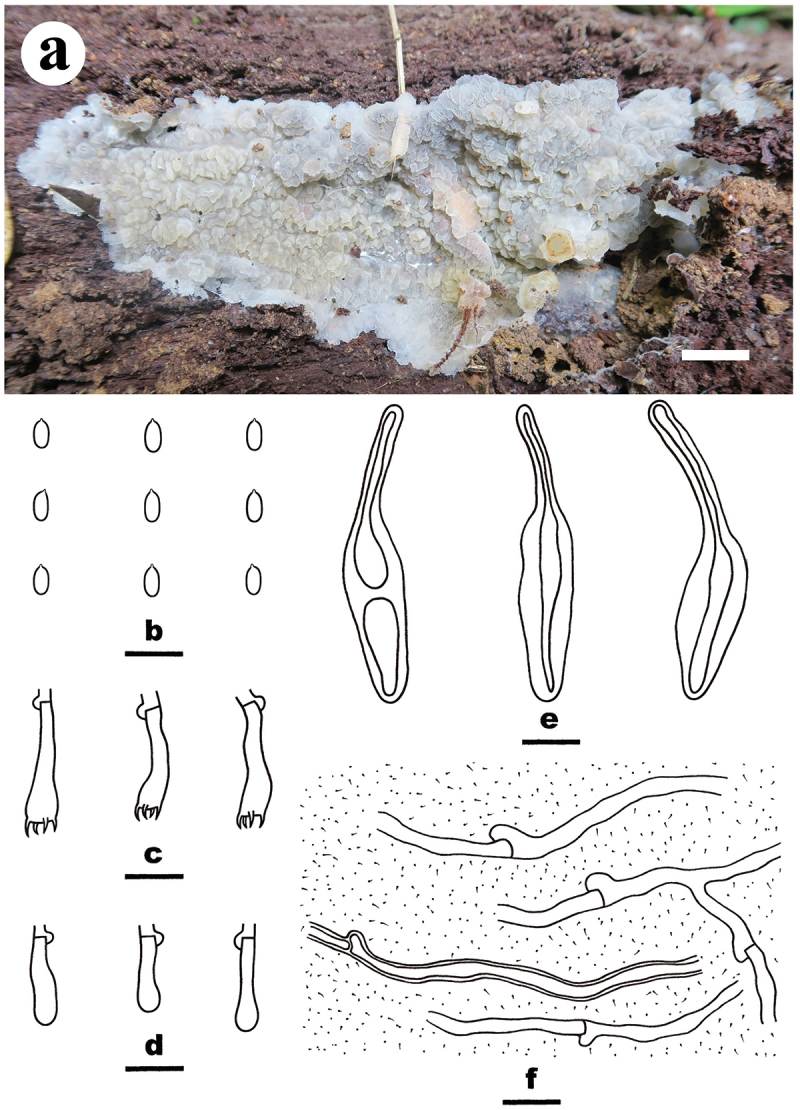
*Meruliella hainanensis* (drawn from the holotype, He 3896). (a) Basidiomes. (b) Basidiospores. (c) Basidia. (d) Basidioles. (e) Cystidia. (f) Hyphae from subiculum. Scale bars: a = 1 cm, b – f = 10 µm.

***Meruliella hainanensis*** S.H. He, Yue Li & Nakasone, **sp. nov**., Figure 6

MycoBank: MB 856589.

*Diagnosis*: The species is recognised by a merulioid hymenophore with a light yellow to greyish orange hymenial surface and fimbriate margin, the presence of subclavate to subfusiform cystidia, and oblong-ellipsoid to subcylindrical basidiospores.

*Type*: China. Hainan Province, Baisha County, Yinggeling Nature Reserve, on fallen angiosperm trunk, 9 June 2016, S.H. He, He 3896 (holotype, BJFC 022398; isotype, CFMR).

*Etymology*: *hainanensis* (Lat.): refers to the type locality, Hainan Province in China.

*Fruiting body*: Basidiomes annual, resupinate, widely effused, closely adnate, inseparable from substrate, ceraceous to subgelatinous, first as small patches, later confluent up to 10.5 cm long, 4.5 cm wide, up to 300 µm thick in section. Hymenophore merulioid, light yellow (4A4) to greyish orange (5B4) when dry, unchanged in KOH, not cracked; margin thinning out, adnate, indistinct, fimbriate, paler than or concolorous with hymenophore surface. Context light yellow.

*Microscopic structures*: Hyphal system monomitic; generative hyphae with clamp connections. Subiculum distinct, thickening; hyphae embedded in a gelatinous matrix, rather sparse, colourless, thin- to slightly thick-walled, smooth, moderately branched, more or less parallel to the substrate, agglutinated, 1.5–5 µm in diam. Subhymenium distinct; hyphae colourless, thin-walled, smooth, infrequently branched, rarely septate, more or less vertical, interwoven, agglutinated, 1–2 µm in diam. Cystidia subclavate to subfusiform, tapering to a long base, colourless, thick-walled, smooth, embedded, or projecting beyond the hymenium up to 30 µm, 36–50 × 7–12 µm. Basidia subcylindrical, slightly sinuous, colourless, thin-walled, smooth, with a basal clamp connection and four sterigmata, 15–18 × 3.5–5 µm; basidioles similar to basidia but smaller. Basidiospores oblong-ellipsoid to subcylindrical, with an apiculus, colourless, thin-walled, smooth, IKI–, CB–, (3.5–) 3.8–4.6 (−4.8) × 1.8–2.2 (−2.5) µm, L = 4.1 µm, W = 2 µm, Q = 2.1 (*n* = 30/1).

*Notes*: *Meruliella hainanensis* is characterised by having ceraceous to subgelatinous basidiomes with merulioid hymenophores, subclavate to subfusiform, thick-walled, smooth cystidia, and oblong-ellipsoid to subcylindrical basidiospores. *Meruliella lindtneri* differs by its conical lamprocystidia, larger basidiospores, and distribution in Europe (5–6.5 × 2.5–3 µm; Eriksson et al. 1981).

***Meruliella lindtneri*** (Pilát) S.H. He, Yue Li & Nakasone, **comb. nov.**

MycoBank: MB 856590.

≡ *Peniophora lindtneri* Pilát, Bull. Trimestriel Soc. Mycol. France 53 (1): 97, 1937.

≡ *Phlebia lindtneri* (Pilát) Parmasto, Wahlenbergia 1: 74, 1975.

≡ *Pseudophlebia lindtneri* (Pilát) C.L. Zhao, J. Fungi 9 (3, no. 320): 33, 2023.

*Notes*: Zhao et al. (2023) placed *Peniophora lindtneri* in *Pseudophlebia* although this lineage was not well-supported. We prefer to treat *Meruliella* and *Pseudophlebia* as separate genera based on morphological and molecular evidence as discussed above. *Meruliella lindtneri* and *M. hainanensis* are closely related sister species that are readily differentiated by cystidia, basidiospore size, and distribution (Figures 1, 2).

***Merulius*** Fr., Systema Mycologicum 1: 326, 1821.

= *Noblesia* Nakasone, Mycological Progress 20 (11): 1489, 2021.

*Type species*: *Merulius tremellosus* Fr., Systema Mycologicum 1: 327, 1821.

*Notes*: *Merulius* is an old name and was once regarded as a synonym of *Phlebia* (Nakasone and Burdsall 1984), but molecular evidence showed that *Phlebia* s.l. is polyphyletic, and *Merulius* s.s. is a distinct genus (Justo et al. 2017; Lira et al. 2022; Zhao et al. 2023). Chen et al. (2021) favoured a broad concept of *Phlebia* to include the species of *Merulius*, *Aurantiopileus*, *Aurantiporus*, and *Pappia*. Based on our phylogenetic analyses (Figures 1, 2), we prefer to treat them as separate genera whereas *Noblesia* Nakasone, typified by *Sistotrema croceum* Schwein., is regarded as a synonym of *Merulius*. *Merulius* formed a well-supported distinct clade including a new species and three new combinations proposed below. Morphologically, *Merulius* is characterised by resupinate, effused-reflexed to pileate, ceraceous to coriaceous basidiomes with smooth, grandinioid, hydnoid, alveolate to poroid hymenophores, monomitic hyphal systems usually with clamp connections, with or without cystidia, and allantoid to ellipsoid basidiospores.

**Figure 7.**
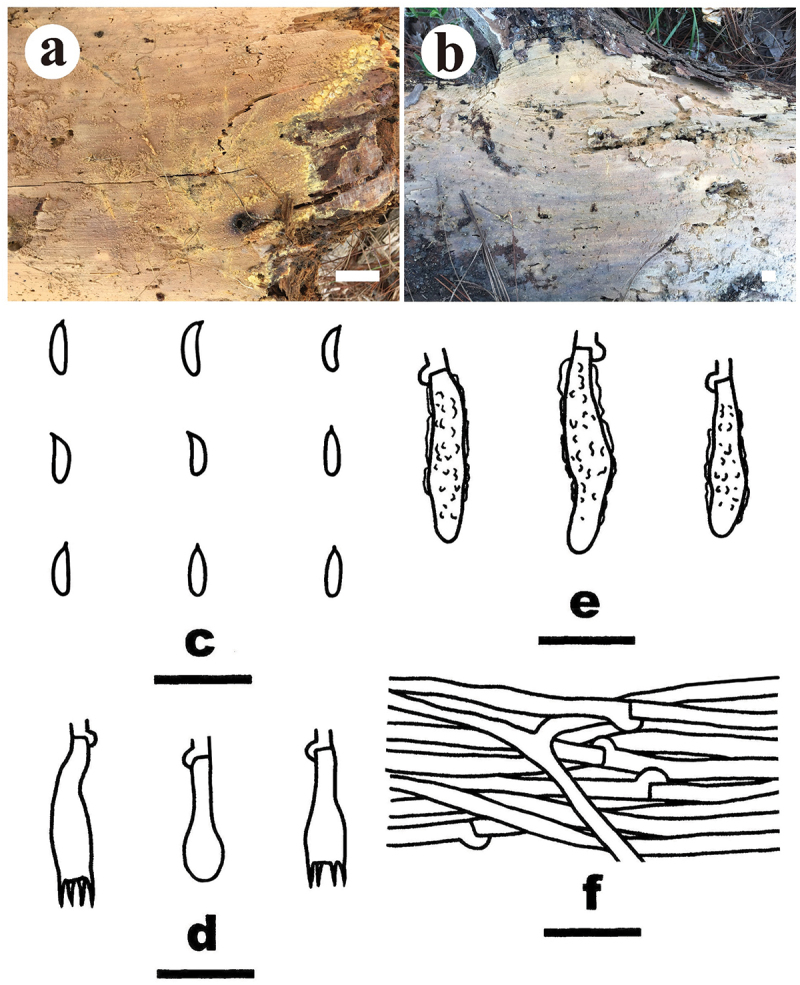
*Merulius pinicola* (drawn from the holotype, He 5267). (a, b) Basidiomes (a He 5267, b He 5273). (c) Basidiospores. (d) Basidia and a basidiole. (e) Cystidia. (f) Hyphae from subiculum. Scale bars: a – b = 1 cm, c – f = 10 µm.

***Merulius pinicola*** S.H. He, Yue Li & Nakasone, **sp. nov**., Figure 7

MycoBank: MB 856591.

*Diagnosis*: The species is recognised by a smooth hymenophore with a greyish orange hymenial surface that darkening in KOH, the presence of subclavate to subcylindrical cystidia, and allantoid to subcylindrical basidiospores.

*Type*: Vietnam. Lam Dong Province, Bi Doup Nui Ba National Park, on fallen *Pinus* trunk, 14 October 2017, S.H. He, He 5267 (holotype, BJFC 024785).

*Etymology*: *pinicola* (Lat.): refers to growing on *Pinus*.

*Fruiting body*: Basidiomes annual, resupinate, widely effused, closely adnate, inseparable from substrate, coriaceous, first as small patches, later confluent up to 30 cm long, 18 cm wide, up to 400 µm thick in section. Hymenophore smooth, greyish orange [6B(4–5)], darkening in KOH, not cracked; margin thinning out, adnate, fimbriate, paler than or concolorous with hymenophore surface. Context greyish orange [6B(4–5)].

*Microscopic structures*: Hyphal system monomitic; generative hyphae with clamp connections. Subiculum thin, with compact texture; hyphae colourless, thin-walled, smooth, infrequently branched, moderately septate, more or less parallel to the substrate, agglutinated, 2–4 µm in diam. Subhymenium distinct, thickening, with dense texture; hyphae colourless, thin-walled, smooth, infrequently branched, rarely septate, more or less vertical, interwoven, agglutinated, 2–2.5 µm in diam. Cystidia subclavate to subcylindrical, colourless, thin-walled, usually with resinous contents, slightly encrusted with tiny, hyaline crystals, 13–21 × 4–5 µm, projecting beyond the hymenium up to 15 µm, also embedded in subhymenium. Basidia clavate, colourless, thin-walled, smooth, with a basal clamp connection and four sterigmata, 10.5–20 × 4–5 µm; basidioles similar to basidia but smaller. Basidiospores allantoid to subcylindrical, with an apiculus, colourless, thin-walled, smooth, IKI–, CB–, (4.1–) 4.2–5 (−5.2) × (1.3–) 1.5–1.8 (−1.9) µm, L = 4.7 µm, W = 1.7 µm, Q = 2.6–3 (*n* = 60/2).

*Additional specimen examined*: Vietnam. Lam Dong Province, Bi Doup Nui Ba National Park, on fallen *Pinus* trunk, 14 October 2017, S.H. He, He 5273 (BJFC 024791).

*Notes*: *Merulius pinicola* is characterised by having smooth, coriaceous hymenophores, subcylindrical cystidia, and allantoid to subcylindrical basidiospores. In the ITS-LSU tree (Figure 2), *M. pinicola* is sister to *M. serialis* that differs in having larger cystidia [50 (−70) × 3.5–5 µm] and basidiospores (5–6 × 1.5–1.8 µm; Eriksson et al. 1981). *Merulius sinensis* C.L. Zhao differs in having grandinoid hymenophores, smooth cystidia, and ellipsoid basidiospores measuring 3.8–4.5 × 2–2.6 µm (Zhao et al. 2023).

***Merulius croceum*** (Schwein.) S.H. He, Yue Li & Nakasone, **comb. nov.**

MycoBank: MB 856592.

≡ *Sistotrema croceum* Schwein., Schriften der Naturforschenden Gesellschaft zu Leipzig 1: 102, 1822.

≡ *Noblesia crocea* (Schwein.) Nakasone, Mycological Progress 20 (11): 1491 (2021).

*Notes*: We adopt a broad concept for *Merulius* and treat *Noblesia* as a synonym. See Nakasone et al. (2021) for more information. *Peniophora femsjoeensis* Litsch. & S. Lundell was also placed in *Noblesia* based on morphology, but we refrain from making the transfer to *Merulius* until molecular data becomes available.

***Merulius leptospermi*** (G. Cunn.) S.H. He, Yue Li & Nakasone, **comb. nov.**

MycoBank: MB 856593.

≡ *Corticium leptospermi* G. Cunn., Transactions and Proceedings of the Royal Society of New Zealand 82 (2), 312.

≡ *Phlebia leptospermi* (G. Cunn.) Stalpers, New Zealand Journal of Botany 23: 307, 1985.

*Notes*: This species has been only reported from New Zealand. It has a smooth, orange to reddish brown, ceraceous hymenophore that turns red in KOH, clavate to capitate cystidia, and ellipsoid basidiospores (Stalpers 1985). Previous studies showed that the species clustered with the *Merulius tremellosus* and other related species with high support values (Figure 2; Chen et al. 2021; Lira et al. 2022; Motato-Vásquez et al. 2022). Thus, we proposed its transfer to *Merulius*.

***Merulius serialis*** (Fr.) S.H. He, Yue Li & Nakasone, **comb. nov.**

MycoBank: MB 856594.

≡ *Thelephora serialis* Fr., Systema Mycologicum 1: 445, 1821.

≡ *Lilaceophlebia serialis* (Fr.) Spirin & Zmitr., Novosti Sistematiki Nizshikh Rastenii 37: 180, 2004.

= *Xerocarpus flavoferrugineus* P. Karst., Hedwigia 34: 8, 1895.

*Notes*: The two samples from USA were nested with the *Merulius* clade and are closely related to *M. pinicola* from Vietnam; morphological differences are discussed above. See Eriksson et al. (1981) for detailed descriptions and illustrations of *M. serialis*.

***Mycoacia*** Donk, Mededelingen van de Nederlandse Mycologische Vereeniging 18–20: 150, 1931.

= *Ceriporiopsis* Domanski, Acta Soc. Bot. Poloniae 32: 731, 1963.

= *Lilaceophlebia* (Parmasto) Spirin & Zmitr., Novosti Sistematiki Nizshikh Rastenii 37: 177, 2004.

*Type species*: *Mycoacia fuscoatra* (Fr.) Donk, Mededelingen van de Nederlandse Mycologische Vereeniging 18–20: 152, 1931.

*Notes*: Zmitrovich (2018) transferred *Poria gilvescens* Bres. and *Corticium lividum* Pers., type species of *Ceriporiopsis* and *Lilaceophlebia* respectively, to *Mycoacia* but didn’t provide the morphological evidence. Figures 1 and 2 and other phylogenetic studies confirm that these genera form a strongly supported, monophyletic clade (Justo et al. 2017; Chen et al. 2021; Lira et al. 2022; Zhao et al. 2023). Based on these phylogenetic studies, the generic concept of *Mycoacia* is broadened to include soft, fleshy to ceraceous basidiomes, on drying becoming crustaceous, corneous, or coriaceous, with smooth, tuberculate, toothed, or poroid hymenophores that often turn red in KOH and with or without cystidia or cystidioles.

**Figure 8.**
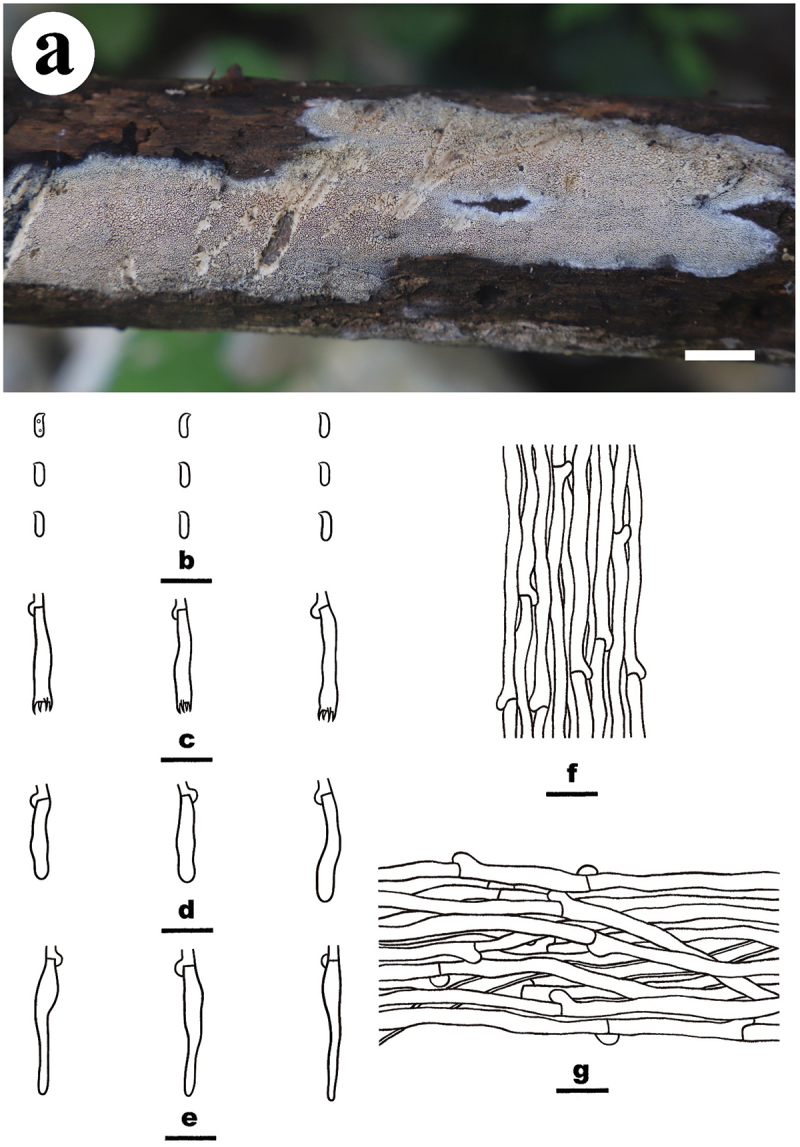
*Mycoacia beijingensis* (drawn from the holotype, He 9275). (a) Basidiomes. (b) Basidiospores. (c) Basidia. (d) Basidioles. (e) Leptocystidia. (f) Hyphae from aculei. (g) Hyphae from subiculum. Scale bars: a = 1 cm, b – g = 10 µm.

***Mycoacia beijingensis*** S.H. He, Yue Li & Nakasone, **sp. nov**., Figure 8

MycoBank: MB 856595.

*Diagnosis*: The species is recognised by a hydnoid hymenophore with an orange to Sahara hymenial surface and fimbriate margin, the presence of subfusiform to subulate leptocystidia, and subcylindrical to suballantoid basidiospores.

*Type*: China. Beijing, Changping District, Baiyanggou Scenic Spot, on fallen angiosperm trunk, 13 August 2024, He 9275 (holotype, BJFC).

*Etymology*: *beijingensis* (Lat.): refers to the type locality in Beijing, China.

*Fruiting body*: Basidiomes annual, resupinate, widely effused, closely adnate, inseparable from substrate, coriaceous, first as small patches, later confluent up to 12.5 cm long, 2.5 cm wide, up to 100 µm thick in section (aculei excluded). Hymenophore hydnoid, orange (6A6) to Sahara (6C5) after dry, slightly darkening in KOH, not cracked; margin thinning out, adnate, fimbriate, white. Aculei conical, fused, 5–15 per mm, 600–1,200 µm long. Context orange.

*Microscopic structures*: Hyphal system monomitic; generative hyphae with clamp connections. Subiculum distinct; hyphae colourless, thin- to slightly thick-walled, smooth, infrequently branched, moderately septate, more or less parallel to the substrate, 1.5–3.8 µm in diam. Aculei hyphae parallel in the core of aculei and often projecting as small fascicles, with clamp connections, colourless, thin-walled, smooth, 2–3 µm in diam. Leptocystidia subfusiform to subulate with tapering apices, colourless, thin-walled, 20–27 × 3–4 µm, projecting beyond the hymenium up to 15 µm. Basidia subcylindrical, sinuous, colourless, thin-walled, smooth, with a basal clamp connection and four sterigmata, 18.5–20 × 3–4 µm; basidioles similar to basidia but slightly smaller. Basidiospores subcylindrical to suballantoid, colourless, thin-walled, smooth, IKI–, CB–, 4–5.5 (−6) × 1.5–2 µm, L = 4.77 µm, W = 1.79 µm, Q = 2.66 (*n* = 30/1).

*Notes*: *Mycoacia beijingensis* is characterised by having hydnoid hymenophores, a monomitic hyphal system with clamped generative hyphae, subfusiform to subulate leptocystidia, and subcylindrical to suballantoid basidiospores. Phylogenetically, *Mycoacia beijingensis* is closely related to *M. aurea* (Fr.) J. Erikss. & Ryvarden (Figure 2). Morphologically, both species have fimbriate margin, clamped generative hyphae, small and similar-sized basidiospores, but *M. aurea* can be distinguished by the longer spines (2–4 mm long) and absence of cystidia (Bernicchia and Gorjón 2010). *Mycoacia subfascicularis* (Wakef.) Hjortstam also resembles *M. beijingensis*, but differs in having larger leptocystidia (27–42 × 3–3.5 µm) and ellipsoid to narrowly ellipsoid basidiospores (4–4.5 × 1.7–2 µm; Chen et al. 2021).

***Mycoacia neotuberculata*** S.H. He, Yue Li & Nakasone, **nom. nov.**

MycoBank: MB 856596.

≡ *Phlebia livida* subsp. *tuberculata* Hallenb. & E. Larss., Mycological Research 97: 353, 1993.

≡ *Phlebia tuberculata* (Hallenb. & E. Larss.) Ghobad-Nejhad, Mycological Progress 11 (1): 32, 2012. nom. illegit., non *P. tuberculata* (Berk. & M.A. Curtis) Tura, Zmitr., Wasser & Spirin.

Non *Mycoacia tuberculata* (Berk. & M.A. Curtis) C.L. Zhao, in Zhao, Qu, Huang & Karunarathna, Journal of Fungi 9(3, no. 320): 24 (2023).

*Notes*: Ghobad-Nejhad and Hallenberg (2012) studied *Phlebia livida* subsp. *livida* (Pers.) Bres. and *P. livida* subsp. *tuberculata* Hallenb. & E. Larss. based on ITS sequences as well as compatibility and isoelectric protein banding patterns (Hallenberg and Larsson 1991), they elevated the latter to the species level. In Chen et al. (2021, Figure 6), samples Chen 3242 and Wu 1708–107 from East Asia and samples MG128 from Spain and FCUG 3157 from Iran formed a lineage correctly named *P. livida* subsp. *tuberculata* [see Ghobad-Nejhad and Hallenberg (2012)]. Chen et al. (2021), however, named this lineage *Stereophlebia tuberculata* (Berk. & M.A. Curtis) Zmitr. *Stereophlebia* was established by Zmitrovich (2018) for *Grandinia tuberculata* Berk. & M.A. Curtis that is quite different morphologically from *P. livida* subsp. *tuberculata* and wasn’t sequenced and phylogenetically studied. Later, Zhao et al. (2023) renamed this lineage *Mycoacia tuberculata* (Berk. & M.A. Curtis) C.L. Zhao. We propose the name *Mycoacia neotuberculata* for this species.

***Mycoaciella*** J. Erikss. & Ryvarden, The Corticiaceae of North Europe 5: 901, 1978.

*Type species*: *Mycoaciella bispora* (Stalpers) J. Erikss. & Ryvarden, The Corticiaceae of North Europe 5: 902, 1978.

*Notes*: Two species of *Mycoaciella* including the type formed a well-supported distinct lineage (Figures 1, 2; Chen et al. 2021; Liu et al. 2022). Morphologically, *Mycoaciella* is characterised by having hydnoid hymenophores, mostly a dimitic or trimitic hyphal system with clamped or simple septate generative hyphae, skeletal hyphae and sometimes microbinding hyphae, and subglobose, ellipsoid to broadly cylindrical basidiospores (Eriksson et al. 1978; Hjortstam and Ryvarden 2004, 2009). Recently, Zhao et al. (2023) described *M. brunneospina* from Yunnan Province, southwestern China, but the type, Zhao 15876, clustered with the type of *M. efibulata* (WEI 16–167) in Figure 2; thus, we place the former in synonymy with *M. efibulata* C.C. Chen & Sheng H. Wu. The distribution of this species is expanded from Yunnan and Taiwan to Beijing, Fujian, and Guizhou Provinces also based on our collection data; see Chen et al. (2021) for more information. In addition, Zhao et al. (2023) proposed a new combination, *Mycoaciella uda* (Fr.) C.L. Zhao, however, this was probably an oversight because this sample clustered with *Crustodontia* and *Luteoporia* and not the *Mycoaciella* lineage; see Figure 1 in Zhao et al. (2023).

***Odoria*** V. Papp & Dima, Mycological Progress 17 (3): 323, 2017.

*Type species*: *Odoria alborubescens* (Bourdot & Galzin) V. Papp & Dima, Mycological Progress 17 (3): 323, 2017.

*Notes*: Papp and Dima (2017) established this monotypic genus for *Phaeolus alborubescens* Bourdot & Galzin, and our phylogenetic analyses support *Odoria* as a distinct genus (Figures 1, 2). It holds a basal position in the clade with other poroid genera such as *Geesterania*, *Luteoporia*, and *Phlebiporia*. Morphologically, *Odoria* is characterised by having pileate basidiomes with white to cream pore surface, round to angular pores, a monomitic hyphal system with clamped generative hyphae, the absence of cystidia, and ellipsoid basidiospores. It resembles *Aurantiporus* by having clamped generative hyphae, the absence of cystidia, and broadly ellipsoid basidiospores, however, the latter genus differs in having pale pink, dark orange, brown to reddish brown pore surface (Papp and Dima 2017).

***Pappia*** Zmitr., Folia Cryptog. Petropol. 6: 101, 2018.

*Type species*: *Pappia fissilis* (Berk. & M.A. Curtis) Zmitr., Folia Cryptog. Petropol. 6: 101, 2018.

*Notes*: *Pappia* includes two taxa but, unfortunately, sequence data for *P. longitubus* (Lespiault) Maffert are not available. Phylogenetically, *Pappia* is basally positioned in the clade with *Phlebia* and poroid genera *Aurantiopileus* and *Aurantiporus* (Chen et al. 2021; Zhao et al. 2023; Figures 1, 2). Many taxa in this clade have basidiomes with orange and cream coloured basidiomes and ellipsoid basidiospores. Morphologically, *Odoria alborubescens* is most similar to *Pappia fissilis*, but differs in having more hirsute pileus, brownish orange to reddish reaction in KOH, and lacking cyanophilous chlamydospores (Papp and Dima 2017).

***Phlebia*** Fr., Systema Mycologicum 1: 426, 1821.

*Type species*: *Phlebia radiata* Fr., Systema Mycologicum 1: 427, 1821.

*Notes*: With the advent of molecular phylogenetics, many *Phlebia* species were transferred to other genera (Chen et al. 2021; Lira et al. 2022; Motato-Vásquez et al. 2022; Zhao et al. 2023). In our analyses, six species are included in the *Phlebia* s.s. clade with moderate to high support values in the ITS-LSU tree (Figure 2) and the multi-gene tree (Figure 1). Morphologically, most species in *Phlebia* s.s. have a monomitic hyphal system with clamped generative hyphae, narrowly clavate basidia, and allantoid, ellipsoid to cylindrical basidiospores. Within the clade, *Phlebia floridensis* Nakasone & Burds. is distinct by having simple-septate generative hyphae, whereas other species have clamp connections and are similar to each other in overall morphology (Nakasone and Burdsall 1995).

***Phlebicolorata*** C.L. Zhao, J. Fungi 9 (3, no. 320): 31, 2023.

*Type species*: *Phlebicolorata brevispora* (Nakasone) C.L. Zhao, J. Fungi 9 (3, no. 320): 32, 2023.

*Notes*: *Phlebicolorata* was established by Zhao et al. (2023) to accommodate *Phlebia brevispora* (type species) and four poroid species, namely, *P. alboaurantia*, *P. crocea*, *P. pseudoplacenta*, and *P. rosea* (C.L. Zhao & Y.C. Dai) C.L. Zhao, that were in *Aurantiporus*. However, as discussed under *Aurantiopileus*, we keep *Phlebicolorata* and *Aurantiporus* as separate genera based on current morphological and molecular evidence. Morphologically, the genus is characterised by having ceraceous to gelatinous basidiomes with tuberculate to hydnoid hymenophore, a monomitic hyphal system with clamped generative hyphae, tubular to obclavate cystidia, and ellipsoid to short cylindrical basidiospores (Zhao et al. 2023).

***Phlebicolorata austroasiana*** (Z.B. Liu & Y.C. Dai) S.H. He, Yue Li & Nakasone, **comb. nov.**

MycoBank: MB 856597.

≡ *Phlebia austroasiana* Z.B. Liu & Y.C. Dai, Journal of Fungi 8 (5, no. 501): 9, 2022.

*Specimen examined*: Sri Lanka. Western Province, Ingiriya, Dombagaskanda Forest Reserve, on *Pinus* trunk, 27 February 2019, S.H. He, He 5744 (BJFC 030611).

*Notes*: Because *P. austroasiana* formed a strongly supported lineage with *P. brevispora* in Figures 1 and 2, we propose to transfer the former to *Phlebicolorata*. See Liu et al. (2020) for detailed descriptions and illustrations of *P. austroasiana*.

***Phlebiodontia*** Motato-Vásq. & Westphalen, Lilloa 59: 318, 2022.

*Type species*: *Phlebiodontia rajchenbergii* Westphalen & Motato-Vásq., Lilloa 59: 320, 2022.

*Notes*: Motato-Vásquez et al. (2022) erected the genus with *P. rajchenbergii* as the type species and proposed two new combinations, *P. acanthocystis* (Gilb. & Nakasone) Motato-Vásq. & Westphalen and *P. subochracea* (Bres.) Motato-Vásq. & Gugliotta. Although the *Phlebiodontia* clade was not well-supported in our ITS-LSU tree (Figure 2), we recognise it as an independent genus consisting of five species, including two new combinations proposed below, and one unnamed taxon (*Phlebia* Chen 3678). In contrast, Zhao et al. (2023) accept an inclusive concept of *Hydnophlebia* that includes taxa we include in *Phlebiodontia* as well as *Porophlebia fimbriata*, a distinct lineage in the family. Morphologically, species of *Phlebiodontia* have ceraceous basidiomes and nodose-septate generative hyphae, whereas *Hydnophlebia* has membranous basidiomes and mostly simple-septate hyphae. *Allophlebia* is closely related to *Phlebiodontia*, see discussion above for their morphological differences. Moreover, there are about 65 base pair differences of total 553 (11.8%) between the ITS sequences of the two genera.

***Phlebiodontia caspica*** (Hallenb.) S.H. He, Yue Li & Nakasone, **comb. nov.**

MycoBank: MB 856598.

≡ *Phlebia caspica* Hallenb., Mycotaxon 11 (2): 460, 1980.

≡ *Hydnophlebia caspica* (Hallenb.) C.L. Zhao, J. Fungi 9 (3, no. 320): 19, 2023.

*Notes* – *Phlebia caspica* was originally described from Iran (Hallenberg 1980). An ITS sequence based on FCUG 3159, from Iran, placed *P. capsica* within the *Phlebiodontia* clade (Figure 2). Thus, the transfer of *P*. *caspica* to *Phlebiodontia* is proposed. Morphologically, *Phlebia caspica* is similar to *Phlebiodontia rajchenbergii* Westphalen & Motato-Vásq. by sharing ceraceous basidiomes with pale yellow hymenophores, clamped generative hyphae, and obclavate cystidia (Motato-Vásquez et al. 2022).

***Phlebiodontia fissurata*** (C.L. Zhao) S.H. He, Yue Li & Nakasone, **comb. nov.**

MycoBank: MB 856599.

≡ *Hydnophlebia fissurata* C.L. Zhao, J. Fungi 9 (3, no. 320): 16, 2023.

*Notes*: *Hydnophlebia fissurata* was recently described from Yunnan Province, southwestern China by Zhao et al. (2023). Our phylogenetic analyses showed that it belongs to *Phlebiodontia* rather than *Hydnophlebia* (Figures 1, 2). Morphologically, *P. fissurata* shares features with other *Phlebiodontia* species such as a ceraceous basidiome, grandinioid hymenophore, and clamped hyphae.

***Phlebiporia*** Jia J. Chen, B.K. Cui & Y.C. Dai, Mycological Progress 13: 568, 2014.

*Type species*: *Phlebiporia bubalina* Jia J. Chen, B.K. Cui & Y.C. Dai, Mycological Progress 13: 569, 2014.

*Notes*: The concept of *Phlebiporia* is expanded to include resupinate basidiomes with poroid and grandinioid hymenophores, monomitic or dimitic hyphal systems with clamped or simple-septate, dextrinoid generative hyphae, thin-walled quasi-binding hyphae in the subiculum, the absence of cystidia, and ellipsoid basidiospores (Chen and Cui 2014). In Figures 1 and 2, two additional taxa joined *P. bubalina* but the lineage was not well-supported. We propose the transfer of the two taxa below to *Phlebiporia* despite differences in basidiome texture, hymenophore configuration, and hyphal septation from the original description of the genus. *Phlebiporia* is closely related to *Geesterania* phylogenetically but distinct by morphology; see discussion under *Geesterania*.

**Figure 9.**
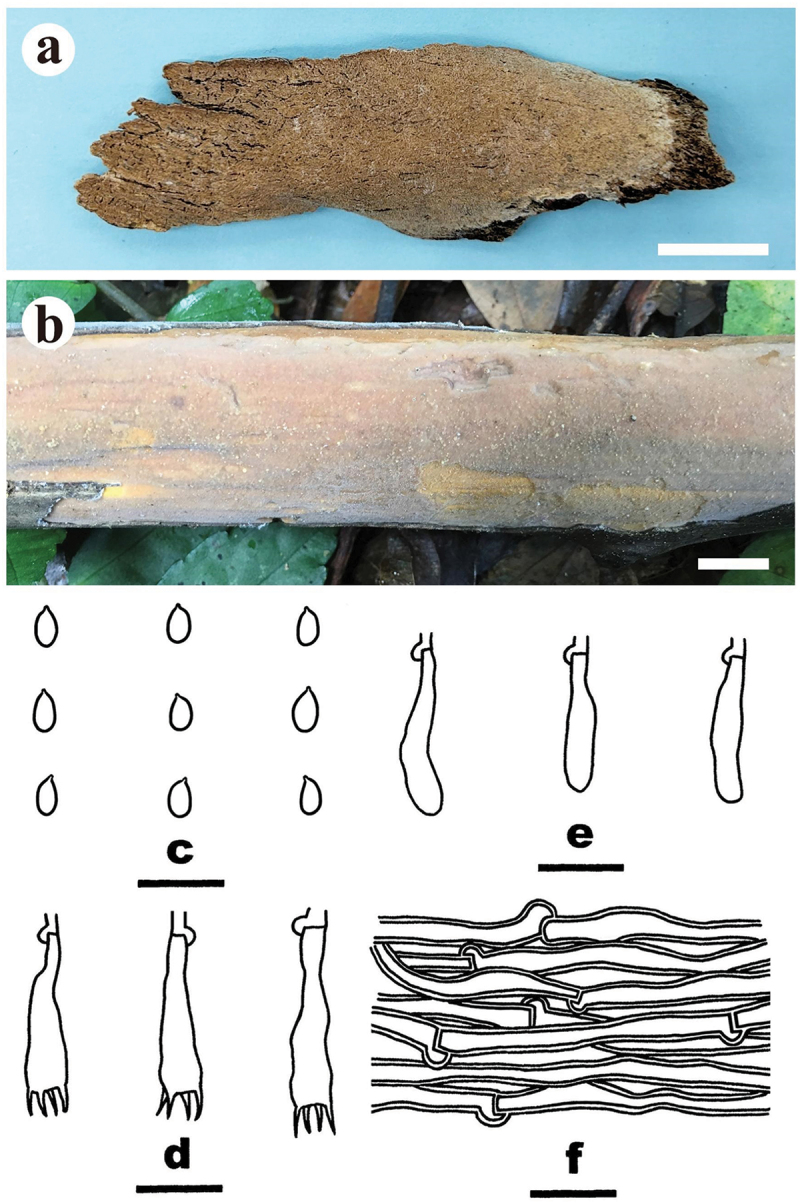
*Phlebiporia crystallifera* (drawn from the holotype, He 20120720–8). (a, b) Basidiomes (a He 20120720–8, b He 5227). (c) Basidiospores. (d) Basidia. (e) Basidioles. (f) Hyphae from subiculum. Scale bars: a – b = 1 cm, c – f = 10 µm.

***Phlebiporia crystallifera*** S.H. He, Yue Li & Nakasone, **sp. nov**., Figure 9

MycoBank: MB 856600.

*Diagnosis*: The species is recognised by a grandinioid hymenophore with a greyish orange and cracked hymenial surface that slightly darkening in KOH, the absence of hyphidia and cystidia, and ellipsoid basidiospores.

*Type*: China. Guangxi Autonomous Region, Longzhou County, Nonggang Nature Reserve, on fallen angiosperm trunk, 20 July 2012, S.H. He, He 20120720–8 (holotype, BJFC 014480).

*Etymology*: *crystallifera* (Lat.): refers to the abundant crystal masses in the basidiome trama.

*Fruiting body*: Basidiomes annual, resupinate, widely effused, closely adnate, inseparable from substrate, ceraceous to crustaceous, first as small patches, later confluent up to 12.5 cm long, 3 cm wide, up to 200 µm thick in section (aculei excluded). Hymenophore grandinioid from protruding crystal masses, greyish orange [6B(4–5)], slightly darkening in KOH, deeply cracked after drying; margin thinning out, adnate, indistinct, paler than or concolorous with hymenophore surface. Context greyish orange [6B(4–5)].

*Microscopic structures*: Hyphal system monomitic; generative hyphae with clamp connections, dextrinoid, CB–. Crystal masses abundant throughout trama. Subiculum distinct, with a dense texture; hyphae colourless, thick-walled, smooth, infrequently branched, moderately septate, more or less parallel to the substrate, agglutinated, 2–4 µm in diam. Subhymenium distinct, thickening, with dense to somewhat compact texture; hyphae colourless, slightly thick-walled, smooth, infrequently branched, rarely septate, more or less vertical, interwoven, agglutinated, 2–3 µm in diam. Hyphidia and cystidia absent. Basidia subclavate, slightly sinuous, colourless, thin-walled, smooth, with a basal clamp connection and four sterigmata, 17–25 × 3–5.3 µm; basidioles similar to basidia but smaller. Basidiospores ellipsoid, with an apiculus, colourless, thin-walled, smooth, IKI–, CB–, (3.7–) 3.8–4.8 (−5.1) × (2.1–) 2.2–2.8 (−3) µm, L = 4.2 µm, W = 2.5 µm, Q = 1.5–1.7 (*n* = 60/2).

*Additional specimen examined*: Vietnam. Lam Dong Province, Bi Doup Nui Ba National Park, on dead angiosperm branch, 14 October 2017, S.H. He, He 5227 (BJFC 024745, CFMR).

*Notes*: *Phlebiporia crystallifera* is characterised by its ceraceous basidiome, grandinioid hymenophore, a monomitic hyphal system with clamped generative hyphae, embedded crystal masses, lacking cystidia, and ellipsoid basidiospores. In the phylogenetic trees (Figures 1, 2), *P*. *crystallifera* formed a moderately supported lineage sister to *P. bubalina*, which differs by developing a poroid hymenophore, simple-septate generative hyphae, and quasi-binding hyphae (Chen and Cui 2014). *Luteoporia lutea* is similar to *P*. *crystallifera* by sharing grandinioid-odontoid hymenophores and clamped generative hyphae but differs in having golden yellow basidiomes, cystidia, and thick-walled basidiospores (Cunningham 1959).

**Figure 10.**
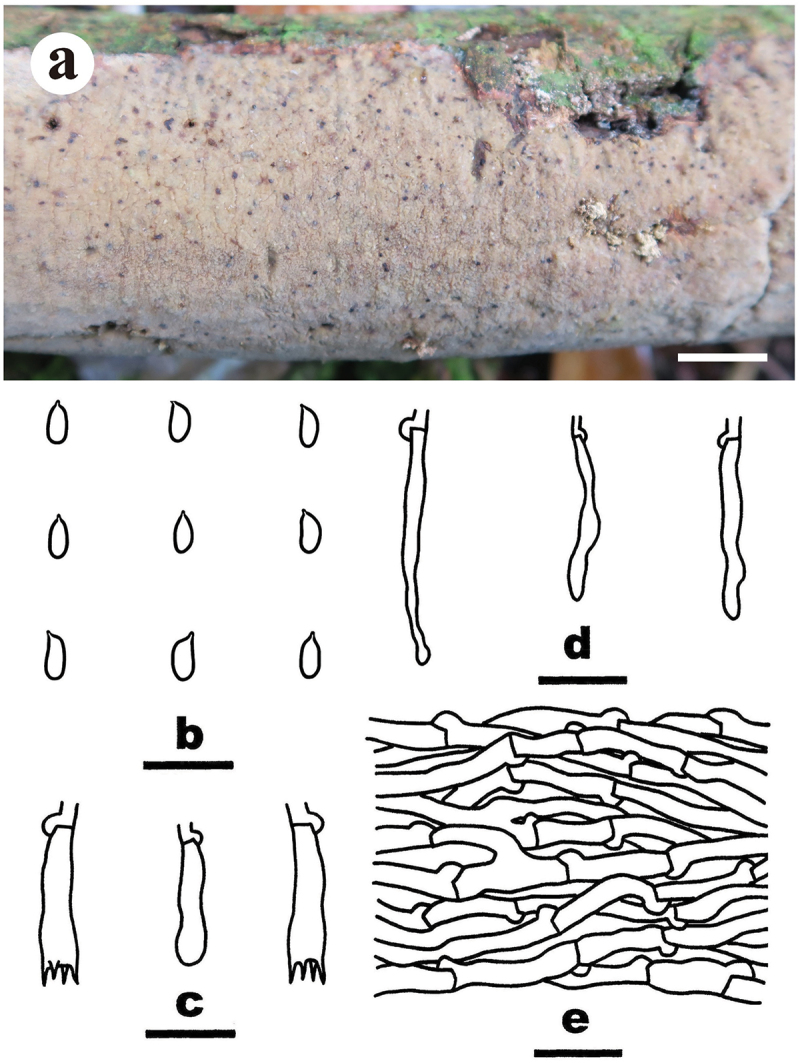
*Phlebiporia odontoidea* (drawn from the holotype, He 4903). (a) Basidiomes. (b) Basidiospores. (c) Basidia and a basidiole. (d) Hyphidia. (e) Hyphae from subiculum. Scale bars: a = 1 cm, b – e = 10 µm.

***Phlebiporia odontoidea*** S.H. He, Yue Li & Nakasone, **sp. nov**., Figure 10

MycoBank: MB 856601.

*Diagnosis*: The species is recognised by an odontoid hymenophore with a greyish orange and cracked hymenial surface that slightly darkening in KOH, the absence of cystidia, and oblong ellipsoid to subcylindrical basidiospores.

*Type*: China. Guangxi Autonomous Region, Jinxiu County, Dayaoshan Nature Reserve, Yinshan Park, on fallen angiosperm trunk, 16 July 2017, S.H. He, He 4903 (holotype, BJFC 024422; isotype, CFMR).

*Etymology*: *odont*-(Greek): refers to the toothed hymenophore.

*Fruiting body*: Basidiomes annual, resupinate, widely effused, closely adnate, inseparable from substrate, ceraceous to crustaceous, first as small patches, later confluent up to 10 cm long, 4.5 cm wide, up to 360 µm thick in section (aculei excluded). Hymenophore odontoid, greyish orange (6B4), slightly darkening in KOH, densely cracked after dry; margin thinning out, adnate, indistinct, concolorous with hymenophore surface. Aculei cylindrical to narrowly conical, with acute sterile apex, separate or laterally fused at base, 6–10 per mm, up to 240 μm long. Context greyish orange (6B4).

*Microscopic structures*: Hyphal system monomitic; generative hyphae with clamp connections, dextrinoid, CB–. Subiculum thin, with a dense texture; hyphae colourless, thin- to slightly thick-walled, smooth, infrequently branched, frequently septate, more or less parallel to the substrate, agglutinated, 2–4.2 µm in diam. Subhymenium distinct, thickening, with a dense to somewhat compact texture; hyphae colourless, thin- to slightly thick-walled, smooth, infrequently branched, rarely septate, more or less vertical, interwoven, agglutinated, 1.5–3 µm in diam. Hyphidia present, sinuous, colourless, thin-walled, smooth, 16–25 × 1.5–2.5 µm. Cystidia absent. Basidia subcylindrical, colourless, thin-walled, smooth, with a basal clamp connection and four sterigmata, 14.5–21 × 3–5 µm; basidioles similar to basidia but smaller. Basidiospores oblong ellipsoid to subcylindrical, with an apiculus, colourless, thin-walled, smooth, IKI–, CB–, (3.9–) 4–4.7 (−4.9) × 2–2.1 (−2.2) µm, L = 4.3 µm, W = 2.1 µm, Q = 2.1 (*n* = 30/1).

*Notes*: *Phlebiporia odontoidea* is characterised by having odontoid hymenophores, clamped generative hyphae, hyphidia, and oblong ellipsoid to subcylindrical basidiospores. In the phylogenetic trees (Figures 1, 2), the type specimen and a sample from Taiwan (Wu 1210–7) formed a distinct lineage sister to the *P. crystallifera* and *P. bubalina* clade. Morphologically, *P*. *crystallifera* differs from *P. odontoidea* by having numerous crystals embedded in trama, slightly wider basidiospores, and lacking hyphidia, whereas *P. bubalina* differs in having poroid hymenophores, simple-septate generative hyphae, and quasi-binding hyphae (Chen and Cui 2014).

***Porophlebia*** S.H. He, Yue Li & Nakasone, **gen. nov.**

MycoBank: MB 856602.

*Diagnosis*: The species is recognised by a poroid hymenophore with a white to cream to clay-pink pore surface when fresh, the absence of cystidia, and oblong-ellipsoid to subcylindrical basidiospores.

*Type species*: *Porophlebia fimbriata* (C.L. Zhao & Y.C. Dai) S.H. He, Yue Li & Nakasone.

*Etymology*: *poro* (Lat.) = poroid + *Phlebia*: similar to the genus *Phlebia*, but with true poroid hymenophores and phylogenetic differences.

Basidiomes annual, resupinate, soft corky when fresh, becoming corky upon drying. Hymenophore poroid, pore surface white to cream to clay-pink when fresh, turning to cinnamon to yellowish-brown upon drying; pores angular, brittle. Margin distinct, white, fimbriate. Hyphal system monomitic; generative hyphae with clamp connections. Cystidia none. Basidia long-clavate to pyriform, with four sterigmata. Basidiospores oblong-ellipsoid to subcylindrical, colourless, thin-walled, smooth, IKI–, CB–. Associated with a white rot of wood.

*Notes*: This genus is difficult to distinguish from species in *Cerioporiopsis* s.l. but phylogenetically, *Porophlebia* is distinct. Presently monotypic, it is characterised by resupinate basidiomes with brittle pores that turn red in KOH, a monomitic hyphal system with clamped generative hyphae, the absence of cystidia and oblong-ellipsoid to subcylindrical basidiospores. In Figures 1 and 2, *Porophlebia* formed a distinct lineage sister to *Allophlebia*, *Hydnophlebia*, and *Phlebiodontia*. However, *Allophlebia* and *Phlebiodontia* have non-poroid hymenophores, whereas *Hydnophlebia* has hyphal strands, simple-septate generative hyphae and the presence of cystidia.

***Porophlebia fimbriata*** (C.L. Zhao & Y.C. Dai) S.H. He, Yue Li & Nakasone, **comb. nov.**

MycoBank: MB 856603.

≡ *Ceriporiopsis fimbriata* C.L. Zhao & Y.C. Dai, in Zhao, Wu, Liu & Dai, Nova Hedwigia 101(3–4): 409, 2015.

*Specimens examined*: China. Hunan Province, Zhangjiajie, Zhangjiajie Forest Park, on fallen angiosperm trunk, 17 August 2010, C.Y. Dai, Dai 11672 (holotype, BJFC 008796; isotype, CFMR); Jiangsu Province, Nanjing, Zijinshan Forest Park, on rotten wood of *Liquidambar*, 3 June 2005, B.K. Cui, Cui 1671 (BJFC 020511, CFMR); Yunnan Province, Kunming, Heilongtan Park, on fallen angiosperm trunk, 2 July 2019, C.Y. Dai, Dai 20020 (BJFC 031694) & C.Y. Dai, Dai 20021 (BJFC 031695); Ruili, Moli Tropical Rainforest Scenic Area, on rotten angiosperm wood, 17 September 2017, B.K. Cui, Cui 16216 (BJFC 029515, CFMR).

*Notes*: The species was described from Hunan and Jiangsu Provinces, and later found also in Yunnan Province. See Zhao et al. (2015) for detailed descriptions and illustrations.

***Pseudophlebia*** C.L. Zhao, J. Fungi 9 (3, no. 320): 33, 2023.

*Type species*: *Pseudophlebia setulosa* (Berk. & M.A. Curtis) C.L. Zhao, J. Fungi 9 (3, no. 320): 33, 2023.

*Notes*: The genus was recently introduced to accommodate *Hydnum setulosum* Berk. & M.A. Curtis, *Peniophora lindtneri* Pilát, *Aurantiopileus mayaensis*, and *Ceriporiopsis semisupina* C.L. Zhao, B.K. Cui & Y.C. Dai (Zhao et al. 2023) despite weakly supported branches in phylogenetic studies (Chen et al. 2021; Zhao et al. 2023; Figures 1, 2). Herein, we retain only the type species, *Hydnum setulosum* in *Pseudophlebia* and include a new species described below. For the other taxa, *P. lindtneri* is the type of *Meruliella*, whereas *A. mayaensis* and *C. semisupina* are in the *Aurantiopileus* lineage. Morphologically, *Pseudophlebia* s.s. is characterised by having ceraceous basidiomes with hydnoid hymenophores, presence of lamprocystidia, and ellipsoid to broadly cylindrical basidiospores (Nakasone and Burdsall 1995). *Meruliella* and *Pseudophlebia* have ceraceous basidiomes, clamped generative hyphae, cystidia, and ellipsoid to cylindrical basidiospores, but the former has alveolate to reticulate hymenophores and a gelatinous subicular matrix where the latter has an odontoid or hydnoid habit.

**Figure 11.**
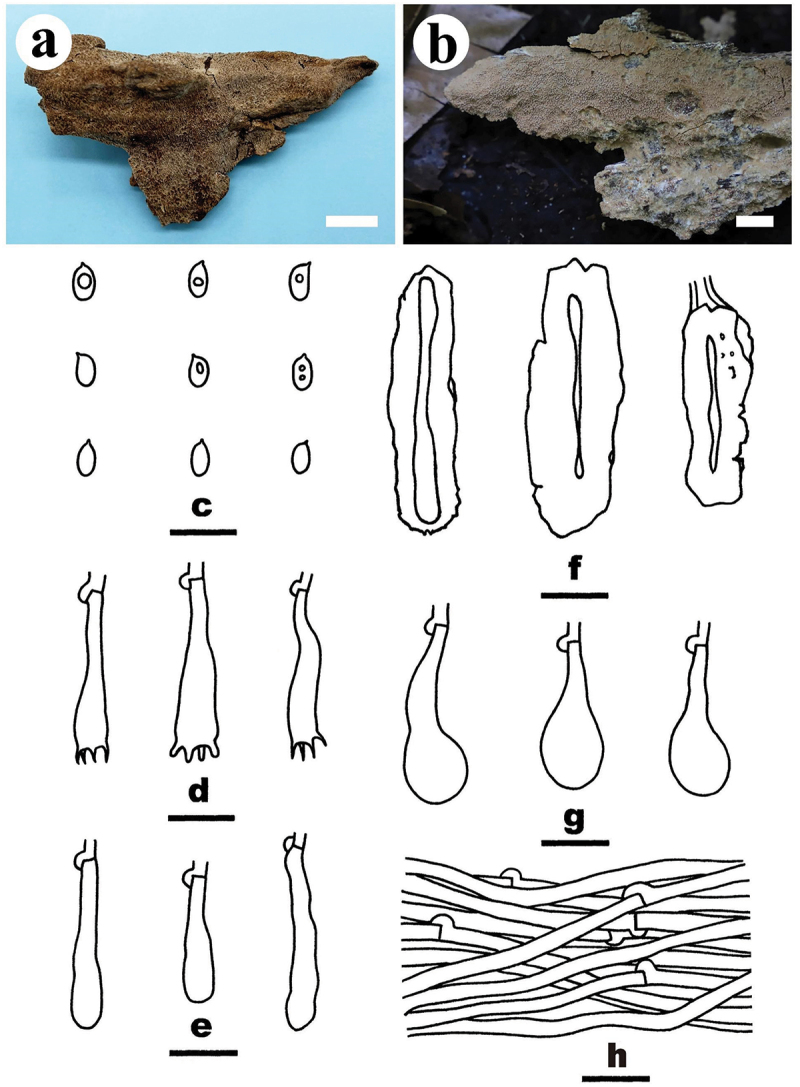
*Pseudophlebia vesiculosa* (drawn from the holotype, He 5730). (a, b) Basidiomes (a He 5730, b He 6403). (c) Basidiospores. (d) Basidia. (e) Basidioles. (f) Lamprocystidia. (g) Vesicular cystidia. (h) Hyphae from subiculum. Scale bars: a – b = 1 cm, c – h = 10 µm.

***Pseudophlebia vesiculosa*** S.H. He, Yue Li & Nakasone, **sp. nov**., Figure 11

MycoBank: MB 856604.

*Diagnosis*: The species is recognised by an odontoid hymenophore with an orange to brownish orange hymenial surface that slightly darkening in KOH, two types of cystidia, and ellipsoid basidiospores.

*Type*: Sri Lanka. Western Province, Ingiriya, Dombagaskanda Forest Reserve, on rotten angiosperm trunk, 27 February 2019, S.H. He, He 5730 (holotype, BJFC 030597).

*Etymology*: *vesiculosa* (Lat.): refers to the vesicular cystidia.

*Fruiting body*: Basidiomes annual, resupinate, widely effused, closely adnate, inseparable from substrate, coriaceous to ceraceous, first as small patches, later confluent up to 10 cm long, 3.5 cm wide, up to 160 µm thick in section (aculei excluded). Hymenophore odontoid, orange (6A6) to brownish orange [6C(5–6)] after drying, slightly darkening in KOH, not cracked; margin thinning out, adnate, indistinct, concolorous with hymenophore. Context orange.

*Microscopic structures*: Hyphal system monomitic; generative hyphae with clamp connections. Subiculum thin; hyphae colourless, thin-walled, smooth, infrequently branched, moderately septate, interwoven, 2–4 µm in diam. Subhymenium distinct, thickening, composed of numerous cystidia and vertical hyphae; hyphae colourless, thin-walled, smooth, infrequently branched, rarely septate, interwoven, agglutinated, 1.5–2 µm in diam. Cystidia two types: (1) lamprocystidia abundant, subcylindrical to subconical, heavily encrusted with crystals, thick-walled, mostly embedded or projecting beyond the hymenium up to 25 µm, 30–105 × 8–13 µm; (2) vesicular, colorless, thin-walled, mostly embedded, 18–30 × 8–10 µm. Basidia subclavate, colorless, thin-walled, smooth, with a basal clamp connection and four sterigmata, 19–23 × 3–6 µm; basidioles similar to basidia but smaller. Basidiospores ellipsoid, with an apiculus, colorless, thin-walled, smooth, usually with oil-drops, IKI–, CB–, (3.6–) 3.8–5 (−5.5) × (2.2–) 2.4–3.1 (−3.2) µm, L = 4.3 µm, W = 2.7 µm, Q = 1.6 (*n* = 60/2).

*Additional specimen examined*: Malaysia. Kuala Lumpur, Kota Damansara Community Forest Reserve, on dead angiosperm branch, 6 December 2019, S.H. He, He 6403 (BJFC 033347).

*Notes*: *Pseudophlebia vesiculosa* is characterised by having an odontoid hymenophore, lamprocystidia, vesicular cystidia, and ellipsoid basidiospores. In the phylogenetic trees (Figures 1, 2), *P*. *vesiculosa* and *P. setulosa* form a strong lineage. The latter species differs in having light orange basidiomes with longer teeth, and larger, ellipsoid to broadly cylindrical basidiospores (5–6.5 × 3–3.5 µm) and lacking vesicles (Nakasone and Burdsall 1995).

***Sarcodontia*** Schulzer, Verhandlungen der Zoologisch-Botanischen Gesellschaft Wien 16: 41, 1866.

*Type species*: *Sarcodontia setosa* (Pers.) Donk, Mycologia 44 (2): 262, 1952.

*Notes*: Based on morphological and molecular evidence, Nakasone et al. (2021) emended *Sarcodontia* to restrict it to three species. The phylogenetic relationship between *S. uda* (Fr.) Nikol. and the core lineage of *S. setosa* (Pers.) Donk and *S. amplissima* (Berk. & M.A. Curtis) Nakasone is weak. Similar results were obtained by others (Figures 1, 2; Justo et al. 2017; Chen et al. 2021), and we believe that it is prudent to keep *S. uda* in *Sarcodontia* until more evidence becomes available. Morphologically, *Sarcodontia* is characterised by having ceraceous, yellow basidiomes with hydnoid hymenophores, a monomitic hyphal system with clamped generative hyphae, fusiform cystidia, and cylindrical to broadly ellipsoid basidiospores (Nakasone et al. 2021).

***Scopuloides*** (Massee) Höhn. & Litsch., Wiesner Festschrift: 57: 58, 1908.

*Type species*: *Scopuloides hydnoides* (Cooke & Massee) Hjortstam & Ryvarden, Mycotaxon 9: 509, 1979.

*Notes*: In previous phylogenetic studies (Justo et al. 2017; Chen et al. 2021; Lira et al. 2022; Motato-Vásquez et al. 2022; Zhao et al. 2023) and herein (Figures 1, 2), *Scopuloides* formed a strongly supported monophyletic clade within the Meruliaceae. Morphologically, it is characterised by having ceraceous to crustaceous basidiomes with grandinioid to hydnoid hymenophores, simple-septate generative hyphae, lamprocystidia, and other cystidial elements, and allantoid to ellipsoid basidiospores (Bernicchia and Gorjón 2010; Chen et al. 2021). Phylogenetically (Figure 1), it is sister to *Climacodon* that is very different morphologically with its large, pileate basidiomes with teeth; see discussion above.

**Figure 12.**
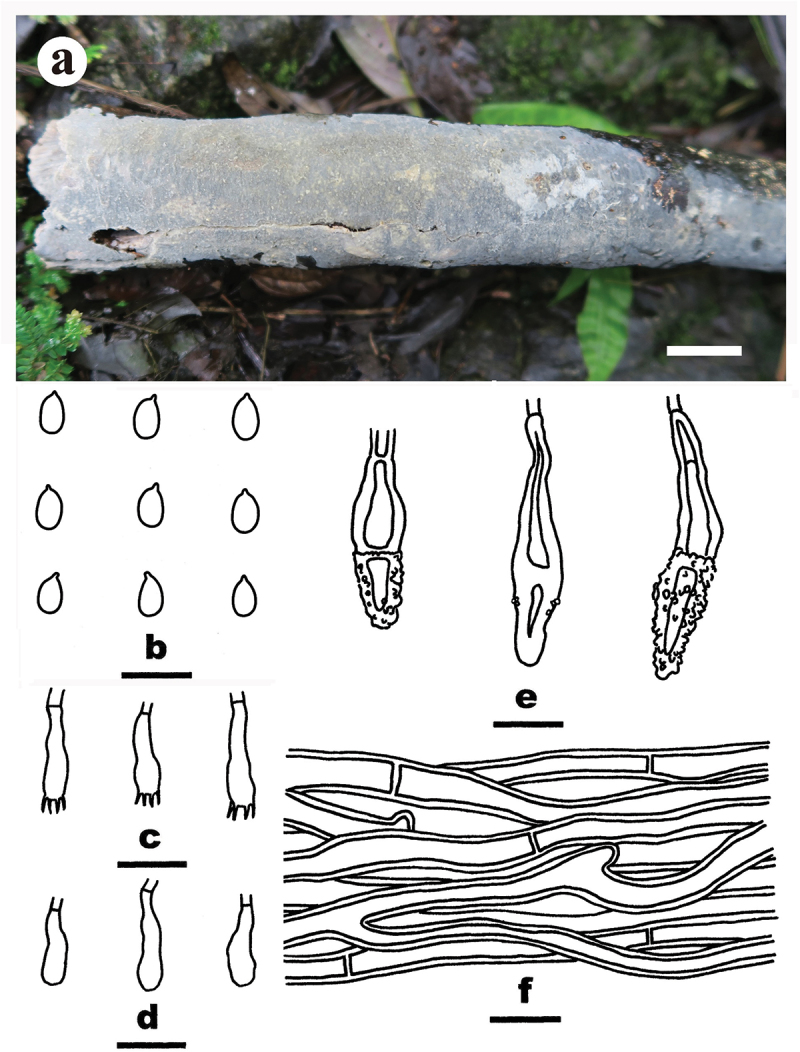
*Scopuloides ellipsoidea* (drawn from the holotype, He 4760). (a) Basidiomes (He 4760). (b) Basidiospores. (c) Basidia. (d) Basidioles. (e) Lamprocystidia. (f) Hyphae from subiculum. Scale bars: a = 1 cm, b – f = 10 µm.

***Scopuloides ellipsoidea*** S.H. He, Yue Li & Nakasone, **sp. nov**., Figure 12

MycoBank: MB 856605.

*Diagnosis*: The species is recognised by a grandinioid hymenophore with a yellowish white to orange grey hymenial surface, the presence of subconical to subfusiform lamprocystidia, and ellipsoid basidiospores.

*Type*: China. Guangxi Autonomous Region, Huanjiang County, Mulun Nature Reserve, on dead angiosperm branch, 10 July 2017, S.H. He, He 4760 (holotype, BJFC 024278).

*Etymology*: *ellipsoidea* (Lat.): refers to the ellipsoid basidiospores.

*Fruiting body*: Basidiomes annual, resupinate, widely effused, closely adnate, inseparable from substrate, ceraceous, first as small patches, later confluent up to 10 cm long, 3.5 cm wide, up to 60 µm thick in section (aculei excluded). Hymenophore grandinioid, yellowish white (4A2), grey (4B1), yellowish grey (4B2) to orange grey (5B2), unchanged in KOH, not cracked; margin thinning out, adnate, indistinct, concolorous with hymenophore surface. Context grey (4B1).

*Microscopic structures*: Hyphal system monomitic; generative hyphae simple-septate. Subiculum distinct; hyphae colourless, thick-walled, smooth, rarely branched, frequently septate, interwoven, 3–5 µm in diam. Subhymenium thickening; hyphae colourless, slightly thick-walled, smooth, infrequently branched, rarely septate, more or less vertical, interwoven, agglutinated, 1.5–2.5 µm in diam. Lamprocystidia abundant, subconical to subfusiform, colourless, thick-walled, usually heavily encrusted in apical part, mostly embedded, 18–30 × 4.5–11 µm (with encrustation). Basidia subclavate to subcylindrical, colourless, thin-walled, smooth, with a basal simple septum and four sterigmata, 10–18 × 3–4 µm; basidioles similar to basidia but smaller. Basidiospores ellipsoid, with an apiculus, colourless, thin-walled, smooth, IKI–, CB–, (2.4–) 2.6–3.1 (−3.8) × (1.4–) 1.5–1.8 (−1.9) µm, L = 2.9 µm, W = 1.7 µm, Q = 1.7 (*n* = 60/2).

*Additional specimens examined*: China. Guangxi Autonomous Region, Tianlin County, Cenwanglaoshan National Nature Reserve, on dead angiosperm branch, 8 July 2017, S.H. He, He 4681 (BJFC 024200); Yunnan Province, Ruili, Moli Tropical Rainforest Scenic Area, on dead *liana* branch, 2 December 2015, S.H. He, He 3489 (BJFC 021886, CFMR). Sri Lanka. Avissawella, Salgala Forest, on rotten angiosperm trunk, 3 March 2019, S.H. He, He 5792 (BJFC 030659).

*Notes*: *Scopuloides ellipsoidea* is characterised by having yellowish white to orange-grey basidiomes with grandinioid hymenophores, subconical to subfusiform lamprocystidia, and ellipsoid basidiospores. In Figure 2, *S. ellipsoidea* is closely related to *Scopuloides dimorpha* (Sang H. Lin & Z.C. Chen) C.C. Chen & Sheng H. Wu, which differs by having thicker basidiomes with distinct spines and cystidial elements (Lin and Chen 1990; Chen et al. 2021). *Scopuloides hydnoides* (Cooke & Massee) Hjortstam & Ryvarden is similar to *S. ellipsoidea* also by sharing grey basidiomes and simple-septate, thick-walled generative hyphae, but differs in having cracked basidiomes, larger lamprocystidia (40–60 × 8–12 µm), and slightly larger, allantoid basidiospores (3.5–4 × 1.8–2 µm; Hjortstam and Ryvarden 1979; Eriksson et al. 1984).

**Figure 13.**
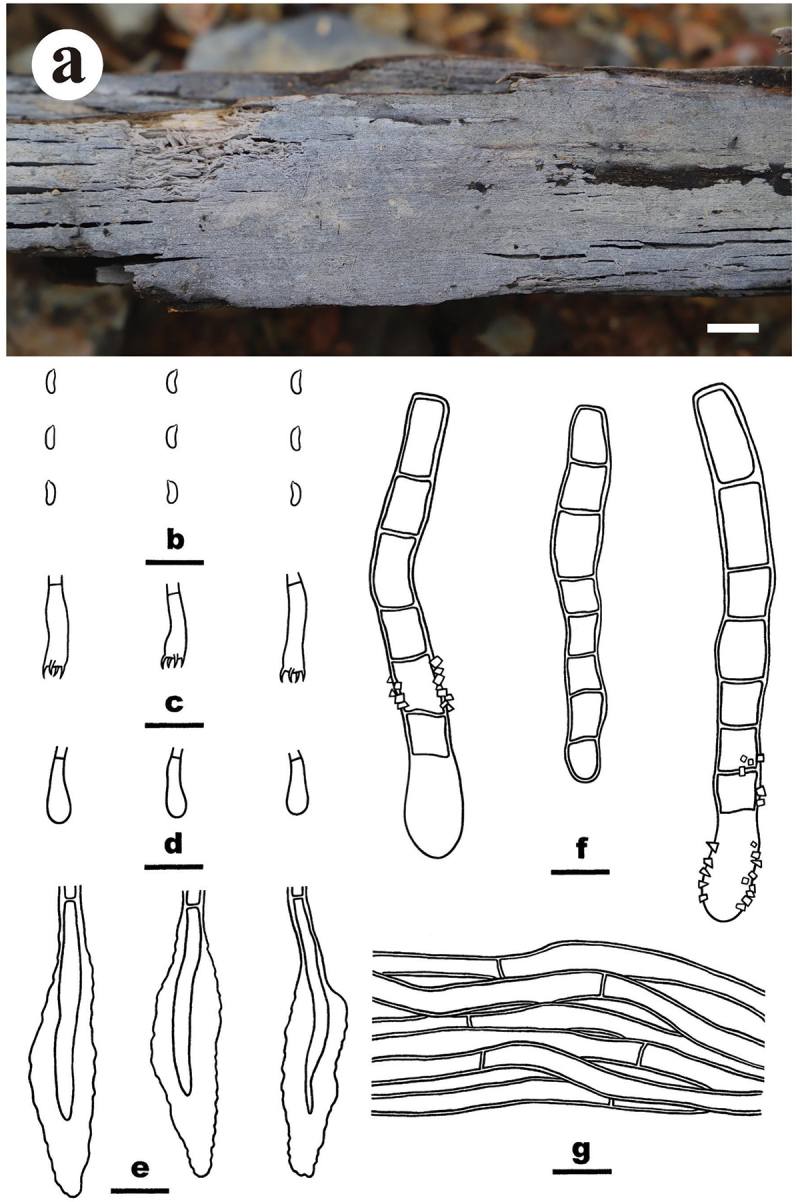
*Scopuloides grandinioides* (drawn from the holotype, He 6295). (a) Basidiomes. (b) Basidiospores. (c) Basidia. (d) Basidioles. (e) Lamprocystidia. (f) Aculeal cystidia. (g) Hyphae from subiculum. Scale bars: a = 1 cm, b – g = 10 µm.

***Scopuloides grandinioides*** S.H. He, Yue Li & Nakasone, **sp. nov**., Figure 13

MycoBank: MB 856606.

*Diagnosis*: The species is recognised by a grandinioid hymenophore with a greyish orange hymenial surface that slightly darkening in KOH, the presence of subulate to subfusiform lamprocystidia, and allantoid to subcylindrical basidiospores.

*Type*: China. Yunnan Province, Xichou County, Xiaoqiaogou Forest Farm, on fallen angiosperm trunk, 16 November 2019, S.H. He, He 6295 (holotype, BJFC 033239).

*Etymology*: *grandinioides* (Lat.): refers to the grandinioid hymenophore.

*Fruiting body*: Basidiomes annual, resupinate, widely effused, closely adnate, inseparable from substrate, ceraceous, first as small patches, later confluent up to 10 cm long, 3 cm wide, up to 40 µm thick in section (aculei excluded). Hymenophore grandinioid, greyish orange (5B4) after dry, slightly darkening in KOH, not cracked; margin thinning out, adnate, indistinct, concolorous with hymenophore surface. Context greyish orange (5B4).

*Microscopic structures*: Hyphal system monomitic; generative hyphae simple-septate. Subiculum thin; hyphae colourless, thick-walled, smooth, rarely branched, frequently septate, more or less parallel to the substrate, 3–5 µm in diam. Subhymenium thickening; hyphae colourless, slightly thick-walled, smooth, infrequently branched, rarely septate, more or less vertical, interwoven, agglutinated, 1.5–2 µm in diam. Lamprocystidia abundant, subulate to subfusiform, colourless, thick-walled, heavily encrusted, embedded, or projecting beyond the hymenium up to 20 µm, 45–55 × 7–14 µm (with encrustation). Aculeal cystidia single, in centre of aculei, cylindrical, colourless, slightly thick-walled, usually apex thin-walled and slightly encrusted, with several secondary septa, 52–90 × 6–12 µm, projecting beyond the hymenium up to 50 µm. Basidia subcylindrical, colourless, thin-walled, smooth, with a basal simple septum and four sterigmata, 10–15 × 3–4 µm; basidioles similar to basidia but smaller. Basidiospores allantoid to subcylindrical, with an apiculus, colourless, thin-walled, smooth, IKI–, CB–, 3–3.5 × (1–) 1.1–1.2 (−1.5) µm, L = 3.2 µm, W = 1.2 µm, Q = 2.8 (*n* = 30/1).

*Notes*: *Scopuloides grandinioides* is characterised by having greyish orange basidiomes with grandinioid hymenophores, subulate to subfusiform lamprocystidia, cylindrical, septate aculeal cystidia, and allantoid to subcylindrical basidiospores. In the phylogenetic tree (Figure 2), the type specimen and two samples from USA formed a well-supported lineage sister to *S. allantoidea* C.C. Chen & Sheng H. Wu and *S. rimosa* (Cooke) Jülich, both differ in having odontoid hymenophores and larger basidiospores (4–4.5 × 1.7–2 µm for *S. allantoidea* in Chen et al. 2021; 4.5–5 × 2–2.5 µm for *S. rimosa* in; Burdsall 1985).

## 4. Discussion

Previously, 28 genera were accepted in Meruliaceae (Chen et al. 2021; Nakasone et al. 2021; Lira et al. 2022; Motato-Vásquez et al. 2022; Zhao et al. 2023). Based on our phylogenetic analyses, three genera, *Ceriporiopsis*, *Lilaceophlebia*, and *Noblesia* were reduced to synonyms of *Mycoacia*. *Stereophlebia* is regarded as genus *incertae sedis*, because the type sequences are unavailable. Herein we accept 26 genera including two new genera, *Meruliella* and *Porophlebia*, in Meruliaceae. Two single-species lineages (“*Phlebia coccineofulva*” and “*Mycoacia* cf. *kurilensis*”) remain unnamed because of insufficient evidence. Furthermore, based on current and revised generic delimitations herein, 11 new combinations in seven genera, and one new name, *Mycoacia neotuberculata*, are proposed. In addition, ten new species in eight genera are described and illustrated based on specimens collected from East Asia, viz. China, Malaysia, Sri Lanka and Vietnam.

In the phylogenetic analysis of the present study (Figures 1, 2), 21 genera showed high statistical supports, including monotypic (*Aurantiopileus*, *Aurantiporus*, *Crustodontia*, *Hydnophlebia*, *Luteoporia, Merulius*, *Mycoacia*, *Phlebia* s.s. and *Scopuloides*) or those with fewer than three species (*Ceriporiopsoides*, *Climacodon*, *Geesterania*, *Hermanssonia*, *Hydnophanerochaete*, *Meruliella*, *Mycoaciella, Odoria, Pappia*, *Phlebicolorata*, *Porophlebia*, and *Pseudophlebia*). We strived to minimise the number of monotypic and small genera and chose to treat *Allophlebia*, *Luteochaete*, *Phlebiodontia*, *Phlebiporia*, and *Sarcodontia* broadly although these lineages are not strongly supported in our phylogenetic trees. The delimitation of these genera may be resolved as more samples from different areas are sequenced and subjected to phylogenetic analyses. Because our generic delimitations are mainly based on phylogenetic analyses of a few genes and gene areas, some genera, such as *Merulius*, *Mycoacia*, and *Phlebiporia*, include species with varied morphological characters. In contrast, some newly created genera, such as *Phlebicolorata*, *Pseudophlebia*, *Meruliella*, and *Hydnophlebia* were segregated from larger genera and have rather uniform morphology.

Recently, many new taxa of Meruliaceae, including the ten new species from this study, were described from East and Southeast Asia (Huang and Zhao 2020; Liu and Yuan 2020; Huang et al. 2020a, 2020b; Liu et al. 2020, 2022; Chen et al. 2021; Zhao et al. 2023; Zhang et al. 2024). Meruliaceae appears to have high diversity in subtropical and tropical areas so undescribed taxa are expected from unexplored and under-collected countries and regions. Molecular data are indispensable for understanding and delimiting species for morphological features which are often limited in usefulness.

As indicated in previous studies (Zhao et al. 2017; Ji et al. 2022), our molecular clock analyses showed that the divergence times of the Meruliaceae with a mean stem age of 186.71 Mya occurred during the early Jurassic, which coincides with the time of rapid differentiation and dominance of the angiosperms (Ji et al. 2022). Our results appear reasonable for most of the species of this family that occur on angiospermous hosts based on collection data. Among the three families in the phlebioid clade, Meruliaceae appeared earlier than Phanerochaetaceae and Irpicaceae (122.4 Mya). According to our results, the 26 genera diverged with a mean stem age between 44.29 to 169.46 Mya, which occupies Eocene, Paleocene, late Cretaceous, early Cretaceous and middle Jurassic periods. The long range of divergence times could explain the fact of high diversity in the family.

Although two new genera and ten new species are described in the present study, we feel that the species diversity of Meruliaceae is far from resolved. With more investigations carried out and samples sequenced, many new species could be found in some genera, especially the monotypic and small genera. It seems that the macro-morphology such as the hymenophore configurations are not reliable in generic delimitation. More genes or even genome sequences should be used to elaborate a natural and reasonable taxonomic system of Meruliaceae in the future.
